# How content complexity and sensory modality influence student satisfaction in art education

**DOI:** 10.1038/s41598-025-08242-5

**Published:** 2025-07-01

**Authors:** Chenxi Sun, Xinan Zhao, Ningning Chen

**Affiliations:** School of Business Administration, Northeastern University, Shenyang, 110167 Liaoning China

**Keywords:** Art education, Student satisfaction, Construal level theory, Academic content abstraction, Visual emphasis

## Abstract

Art education plays a vital role in preserving cultural heritage and promoting sustainable development. However, growing curricular complexity and abstraction present challenges to student satisfaction, a key metric in educational sustainability. This study investigates how perceived content complexity and modality structure affect student satisfaction in art-related programs, drawing on Construal Level Theory (CLT) to explain the psychological mechanisms involved. Based on aggregated data from 73,368 students across 1524 academic programs in China, the results indicate that higher content complexity significantly reduces satisfaction. Moreover, a lower auditory–visual (A/V) ratio—indicating reduced emphasis on visual input—also decreases satisfaction but moderates the negative effect of content complexity. The findings suggest that cognitive-perceptual mechanisms play a central role in shaping students’ evaluative responses and offer implications for improving curriculum design and institutional strategies in art education.

**Supplementary Information:**

The online version contains supplementary material available at 10.1038/s41598-025-08242-5.

## Introduction

Art plays an essential role in culture, reflecting the history, traditions, and values of a society^1^. The art industry spans visual arts, performance, film, and the creative sector, all contributing significantly to regional economies^2,3^. Art schools drive this progress by fostering talent and advancing arts education, research, and academic exchange. Alongside their artistic mission, art programs in higher education also aim to preserve and expand knowledge. However, with growing curriculum demands, competitive pressures, and intensive teaching methods^4^, students face an increasing workload that may conflict with the field’s emphasis on “freedom.” This raises questions: Does the complexity of art education systems heighten student dissatisfaction? And if the trend toward more complex knowledge structures persists, how might arts disciplines adapt?

Student satisfaction research has traditionally examined broad factors like educational experiences, skill development, and community awareness^5–10^. However, studies now increasingly target satisfaction drivers specific to disciplines, regions, and demographics, emphasizing traits, perceived quality, and perceived value^6,11,12^. Yet, consensus on how knowledge complexity impacts satisfaction remains elusive. Simplifying knowledge structures often boosts perceived value, enhancing clarity and engagement^13–15^, while Increasing the complexity of educational content can enhance abstract thinking, promote knowledge interaction, and foster participants’ creativity, thereby improving perceived satisfaction with the educational system^16–20^.

Construal Level Theory (CLT) may bridge these perspectives by framing satisfaction in terms of psychological distance. CLT divides cognitive perception into high-level (abstract) and low-level (concrete) construals, which shape how students interpret information and goals^21,22^. Applied to student satisfaction, CLT helps explain how features such as content abstraction and modality structure may alter affective evaluations—not through motivation or value alignment, but via perceptual and cognitive processing. While widely used in decision-making and consumer research^23,24^, CLT has yet to be extensively applied to educational satisfaction, particularly in arts education.

Despite CLT’s promise, key gaps remain in student satisfaction research. Existing studies often overlook the unique perspectives of arts-related programs, lack multidimensional analyses of abstract-concrete dynamics, and rely on limited sample sizes. This study addresses these gaps by analyzing satisfaction data from 73,368 students across 1,524 arts-related academic programs in China, using a CLT-informed framework to examine how content complexity and modality relate to satisfaction. Rather than identifying the dominant drivers of satisfaction, this research focuses on how cognitive-perceptual characteristics of course design shape students’ affective responses—an angle that contributes both to theory refinement and to practice-oriented strategies for sustainable development in art education.

## Theory and hypotheses

This study applies Construal Level Theory (CLT) to assess whether factors influencing students’ satisfaction with their academic program are processed at abstract or concrete levels. CLT explains how people perceive events based on psychological distance, influencing the abstractness of their representations^25^. When psychological distance is minimal, representations are concrete; when distance increases, interpretations become more abstract^26^. Accordingly, CLT suggests that close events are viewed concretely, emphasizing details, while distant events appear abstract, framed around values or goals^27^. We adopted CLT because it directly explains how abstract content and sensory modality shape evaluative judgments, which aligns with our focus on content complexity and audiovisual structure. The adoption of CLT is analyzed in more detail in Appendix Sect. 1.

Prior research shows that abstract educational goals can foster deeper student engagement by highlighting universal values and long-term benefits, such as personal development and societal impact^28^. However, two limitations exist: (1) Highly abstract disciplines may introduce stress from increased knowledge demands, perceived detachment from real-world relevance, and uncertainty about future careers^29^. Such side effects may impact satisfaction in art disciplines, where abstraction’s complexities and demands often contrast with the value of “academic freedom”^30^. (2) Concrete education methods provide hands-on experiences and clearer career relevance, potentially alleviating dissatisfaction when abstract education becomes overwhelming^31^.

Research indicates that meeting abstract-level needs ensures satisfaction for events perceived as abstract, while meeting concrete needs enhances satisfaction in concrete contexts^32^. This alignment between abstract-concrete levels and satisfaction, known as “construal fit,” suggests that enhancing either abstract or concrete features accordingly may boost satisfaction^33^.

From the perspective of Construal Level Theory (CLT), the key elements of observation can be summarized into three aspects: environmental characteristics, interaction characteristics, and feedback. Environmental characteristics refer to the psychological distance between the evaluator and the evaluated subject. In the context of this study, this represents the default psychological distance between students and their disciplines. Interaction characteristics pertain to the nature of newly introduced decision-making mechanisms, which, in this study, are operationalized as content complexity and auditory-visual (A/V) ratios. Feedback is represented by student satisfaction in this research.

CLT reconciles these differences by emphasizing that the drivers of satisfaction vary depending on the level of abstraction. Simplistically, one could interpret this as follows: for highly abstract disciplines, abstract elements are likely to enhance satisfaction, whereas concrete elements may be more suitable for disciplines with a focus on concreteness^34^.

For arts disciplines, although no study has definitively classified their environmental characteristics as either concrete or abstract, this paper argues that it is reasonable to conceptualize them as concrete. Compared to other common disciplines, arts education places greater emphasis on the interaction of the five senses, particularly visual and auditory elements^35^. Unlike science and engineering or humanities disciplines, arts disciplines focus more on participants’ direct emotional expression of genuine experiences. This psychological atmosphere aligns closely with a relatively short psychological distance.

While in some contexts, art is often described as highly abstract, such narratives typically refer to a unique, extended quality of the arts. However, the realization of this distinctive abstraction generally relies on non-abstract, everyday interactions. Based on the principles of CLT and educational experience, it can be inferred that concrete interaction forms remain crucial in fostering positive psychological feedback in foundational arts education activities.

Under the framework of CLT, when environmental and interaction characteristics align (i.e., achieve a construal fit), feedback tends to reach a positive state; otherwise, dissatisfaction is more likely. It is important to note that this categorization is a preference specific to this study and does not imply that CLT can only be interpreted through these three elements.

### Hypothesis 1

Higher content complexity in academic programs decreases student satisfaction.

As education becomes more intricate, students face extensive, abstracted knowledge systems, often resulting in heightened cognitive load and lower satisfaction^36,37^. This effect is amplified in arts disciplines, where abstraction may overshadow student autonomy and comprehension^17^.

Based on the principles of CLT, we tentatively classify arts disciplines as being associated with concrete environmental characteristics. This implies that participants place significant emphasis on, and derive satisfaction from, psychological fulfillment within low psychological distance contexts. If Hypothesis 1 is validated, it would further strengthen the applicability of CLT logic and empirically demonstrate that the environmental characteristics of arts disciplines align with a concrete level of abstraction.

### Hypothesis 2

Higher auditory-visual ratios in academic programs correlate with lower student satisfaction.

Auditory and visual interactions are common elements for reducing psychological distance. Unlike other disciplines that often emphasize knowledge interaction modes transcending sensory perception—such as reasoning, association, and memory—the focus on auditory and visual interaction is a distinctive feature of arts disciplines. This distinction drives our interest in analyzing patterns related to auditory-visual elements. On one hand, arts disciplines are uniquely suited for evaluating auditory and visual structures across different academic programs, whereas other disciplines are less amenable to such assessments. Given that this study exclusively focuses on arts disciplines, the selected samples are all drawn from this category. On the other hand, under a more refined classification, auditory and visual elements correspond to different levels of abstraction: visual elements tend to align with concreteness, while auditory elements are associated with abstraction, as supported by existing CLT research^35^. This raises an intriguing question: can CLT logic still effectively explain the observed dynamics under this classification? This question lies at the core of our investigation.

Therefore, Hypothesis 2 extends the logic of Hypothesis 1, proposing that since the environmental characteristics of arts disciplines lean toward concreteness, a higher proportion of auditory elements in a program (e.g., music-related disciplines) suggests that its interaction characteristics lean toward abstraction. This misalignment may hinder the formation of a fit, resulting in relatively lower satisfaction feedback. Conversely, for programs with a higher proportion of visual elements (e.g., visual design-related disciplines), the interaction characteristics are more likely to align with the concrete environment, leading to a better fit and higher student satisfaction.

### Hypothesis 3

The auditory-visual ratio moderates the relationship between content complexity and satisfaction.

Within the framework of Construal Level Theory (CLT), the concept of *construal fit* is both significant and commonly studied^26^. In typical research, the environmental characteristic is fixed, and the interaction characteristic is selected to achieve fit. However, this study raises a somewhat unique question: when both the environmental characteristic (students’ psychological distance from their programs) and the primary interaction characteristic (increasing content complexity) are fixed and do not achieve fit, how should a supplementary interaction characteristic (other educational strategies) be adjusted to create fit? Should it align with the environmental characteristic, or with the primary interaction characteristic?

This practical and theoretically meaningful question has not been adequately addressed in previous CLT research. To explore this, the study incorporates the auditory-visual ratio as a moderating variable in Hypothesis 2, testing whether its moderating effect supports alignment with the environmental characteristic or the primary interaction characteristic. Since both content complexity and the auditory-visual ratio can be uniformly interpreted within the CLT framework in terms of abstraction levels, the moderating effect of the auditory-visual ratio gains a stronger theoretical foundation.

Clarifying this issue not only helps reduce the risk of misdirection in similar real-world decision-making scenarios but also contributes to extending the explanatory power of CLT in broader contexts.

**Fig. 1:**
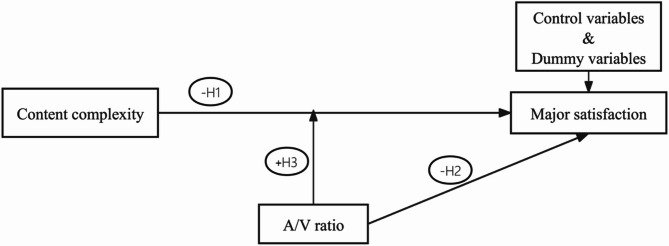
Conceptual model. *Note* A/V ratio represents the abbreviation for the auditory /visual ratio (referring to the auditory-to-visual elements ratio).

These hypotheses are visually summarized in Fig. 1.

## Methods

To examine the impact of the abstraction of educational elements on student satisfaction, a comprehensive, accurate, and real dataset is needed. Previous research mainly relied on experimental studies, interviews, and small-scale questionnaires^7,22,31,32,38^. However, the present study focuses on satisfaction issues related to various programs within the field of arts and requires consideration of heterogeneity effects across different regions^12^. Therefore, a larger and more diverse dataset is necessary for this investigation.

In this study, a nationwide sample within China was used as the foundational dataset. For the dependent variable (program satisfaction), data on students’ satisfaction with their programs were collected from the official website of the Chinese Ministry of Education, known as the " China Higher Education Student Information and Career Center (CHSI)" (https://gaokao.chsi.com.cn/zyk/pub/myd/schAppraisalTop.action, see Table S1 in Appendix). The sample comprises 1,524 independent academic programs across 693 universities in China, covering 23 s-level disciplines within the arts. These programs are distributed across all 31 provinces and 198 cities. A total of 73,368 registered students participated in the satisfaction voting, and each program’s satisfaction score was calculated as the average of individual voting results at the program level.

Regarding the independent variables, information on the complexity of teaching courses and the ratio of audiovisual elements in 23 relevant programs in arts disciplines was obtained from a specialized Chinese college entrance examination advisory company (see Table S1 in Appendix Sect. 2.1) and official websites of multiple universities. Additionally, insights from six arts education experts from various regions of China were consulted. Detailed descriptions of the independent variables will be further elaborated in subsequent sections.

As for the control variables, this study mainly focuses on examining the influence of regional arts-related industry factors on the satisfaction levels of various programs within the universities. We conducted some interesting explorations in this regard, considering that the satisfaction data obtained from CHSI encompassed 1,524 higher education institutions distributed across 198 cities. The business performance in these cities can directly reflect the industrial interactions in the regions where these institutions are located. To this end, we utilized a well-known Chinese corporate information retrieval website, “Tianyancha” (see Table S1 in Appendix Sect. 2.1), to filter companies nationwide in the cultural, sports, and entertainment categories that were operational, with complete contact information, full trademark information, and an employee size of at least 10 people. A total of 9,036 companies met these criteria. By categorizing and aggregating these companies according to their respective cities, we could obtain industry indices for different cities, which could then be assigned to the corresponding universities in those cities.

The study employed a cross-sectional analysis design, and data was collected in April 2024. After processing the statistical information for each program and the industry information in the cities where the universities were located, a dataset of 1,524 valid samples was obtained, containing information on the universities, programs, and geographical locations. All data collection processes were unbiased and free from subjective preferences or discrimination in the selection of different regions, universities, or programs. Thus, the data effectively represented the genuine information of the corresponding indicators. To avoid skewed distributions and nonlinear features, with the exception of the dependent variable, all continuous variables underwent log transformation prior to analysis.

To reduce potential confusion regarding the data, methodology, and analytical procedures employed in this study, we provide a series of detailed supplementary explanations in Appendix. Appendix Sect. 1 elaborates on the theoretical relevance of Construal Level Theory (CLT) to the present research. Appendix Sect. 2 outlines data sources and the operationalization of variables—readers seeking clarification on specific model terms are particularly encouraged to consult Sect. 2.2. Appendix Sect. 3 explains the rationale and procedures for data normalization. Appendix Sect. 4 presents a set of extended model analyses, which may further address questions related to robustness and model specification.

### Variables

#### Dependent variable

The dependent variable in this study is Program satisfaction, defined as the average satisfaction score reported by students within each academic program. Program satisfaction refers to aggregated student satisfaction at the program level, and serves as the outcome variable in all regression models.

The student satisfaction data used in this study were obtained through a survey conducted by the " China Higher Education Student Information and Career Center (CHSI)" and was publicly disclosed. The questionnaire assessed whether the students were satisfied with their programs and whether they would recommend the program to prospective students. This question aligns with the requirements of this study. As the voters were registered and identified current students or graduates, this indicator provided a more balanced representation of overall satisfaction with education and employment compared to other satisfaction-related information.

Each program sample in this study refers to a distinct academic program at a specific university (e.g., Musical Education at Tsinghua University), resulting in 1,524 program-level units of analysis. A total of 73,368 students participated in the voting process, with an average of 48.1 students per program. Each student could only vote once for their respective program. The voting scores were based on the Likert scale (ranging from 1 to 5), and the satisfaction score for each program was calculated as the average of the cumulative voting scores from all participating students. This voting mechanism is consistent with the voting systems used on platforms such as IMDB and Douban movie websites^39^.

Several features of the CHSI system enhance the reliability of the satisfaction scores. First, the data collection is conducted through a real-name authentication system, ensuring that only registered students or graduates can vote. Second, participation is mandatory within the CHSI system, embedded in the academic record verification process. As a result, sampling biases related to region, universitwy tier, or student background (e.g., grade, gender) are significantly reduced. Third, because China’s higher education system operates under a nationally standardized framework, horizontal comparability across universities and regions is high. Together, these features render the dependent variable both reliable and representative for large-scale, cross-institutional analysis. A detailed discussion of the reliability of CHSI data is provided in Appendix Sect. 2.

#### Independent variables

##### Content complexity

Content complexity refers to the degree of complexity within the teaching content of a particular discipline, primarily manifested in the scale of course categories and the number of courses within the curriculum. To comprehensively and systematically outline the complexity of content across 23 arts-related academic programs, we integrated information from three sources: firstly, preliminary measurements of course syllabi were conducted based on internal data from the educational consulting service "Yifan Career Planning System" (see Table S1 in Appendix Sect. 2.1); secondly, supplementary information was gathered from teaching syllabi available on the websites of the Ministry of Education, various universities, and third-party educational consulting websites; thirdly, consultation was sought from six experts in arts education employed at universities across different regions of China, including Northeast (Shenyang, Changchun), Southwest (Guilin), Southeast (Guangzhou), and Central (Beijing, Luoyang). By consolidating information from these three channels, we aimed to ensure the completeness, accuracy, and standard consistency of the data. A detailed explanation of the data sources, evaluation procedures, and reliability assessments for the content complexity variable is provided in Table S2 in Appendix Sect. 2.4.

According to the viewpoint of Construal Level Theory (CLT), when learning a greater variety of courses within a limited timeframe, teachers and students often find it challenging to communicate knowledge details and tend to digest information from a more abstract perspective, thereby leading to content abstraction^25^. Therefore, the number of courses in the curriculum can directly reflect the complexity of the content and also indicate the degree of abstraction specific to the professional content (see Table 1).

**Table 1:** Variable information across 23 disciplines (log-transformed values).

ID	Discipline name	Content	A/V ratio	F/M ratio
1	Music performance	2.944	0.251	0.619
2	Music	2.890	0.288	0.995
3	Theory of composition and composition technology	2.303	0.154	0.447
4	Musical education	2.079	0.182	1.208
5	Recording art	2.303	0.118	0.323
6	Art design	2.890	− 0.588	0.490
7	Visual communication design	2.996	− 0.811	0.663
8	Product design	2.708	− 0.981	0.405
9	Clothing and apparel design	2.303	− 1.099	1.266
10	Public art	2.398	− 0.847	0.532
11	Art and craft	2.773	− 1.253	0.575
12	Digital media arts	2.303	− 0.288	0.405
13	Art and technology	2.996	− 0.470	0.532
14	Performance	1.609	0.251	0.447
15	Theatre	1.792	0.154	0.944
16	Filmology	2.485	− 0.134	1.099
17	Drama film and television literature	2.944	− 0.154	0.944
18	Radio and television director	2.639	− 0.154	0.708
19	Theatrical and film director	2.079	− 0.134	0.323
20	Theatrical film and television art design	2.944	− 0.811	0.619
21	The art of broadcasting and hosting	2.773	0.470	0.708
22	Animation	2.639	− 0.588	0.241
23	Photography and production	2.639	− 0.118	− 0.040

Content represents Content complexity; A/V ratio represents the abbreviation for the auditory /visual ratio (referring to the auditory-to-visual elements ratio); F/M ratio represents the abbreviation for the female-to-male ratio (indicating the gender ratio).

##### Auditory-visual ratio

The auditory-visual ratio is the ratio between the intensity of auditory elements and visual elements in each program. Since the educational content of the same programs in different regions and institutions in China tends to have a certain level of consistency^40^, the information in Table 1 can be inferred to apply to corresponding programs in different universities. Using the auditory-visual ratio variable allows for a more stable description of the auditory-visual structure characteristics of various programs, considering the fluctuation in the mean values of auditory and visual elements across different programs. A detailed explanation of the data sources, evaluation procedures, and reliability assessments for the auditory-visual ratio variable is provided in Table S3 in Appendix Sect. 2.5.

Art-related disciplines are particularly well-suited for evaluation using the audio-visual ratio. This is because these disciplines involve more frequent interactions among the five senses compared to other fields, with a particular emphasis on visual and auditory aspects. Furthermore, the varying focus on either visual or auditory elements across different areas of the arts makes them more distinguishable. These inherent characteristics make art-related disciplines an ideal context for testing the applicability and validity of Construal Level Theory (CLT).

#### Control variables

Due to the distribution of universities across 198 different urban areas in China, regional factors need to be considered as control variables to more accurately estimate the impact of the main explanatory variables on the dependent variable. Therefore, this study’s control variables primarily include the economic interaction status related to the scale and cultural entertainment industry of the regions (cities) where each university is located. The convenience provided by the Tianyancha website (see Table S1 in Appendix Sect. 2.1) facilitates statistical analysis of the economic conditions of enterprises in various cities.

##### Municipality directly under the central government

The special political status of China’s capital and direct-controlled municipalities (four cities in total) is considered a potential influence on satisfaction. Therefore, we include whether the university is located in a direct-controlled municipality as a control variable, including Beijing, Tianjin, Shanghai, and Chongqing.

##### Types of colleges

Chinese universities are usually supervised and funded by relevant government departments, and the level of supervision may have an impact on satisfaction. We use “Department level” as a control variable, representing the level of affiliated department: Central or ministries = 0, provincial departments = 1, municipal departments = 2.

##### Firm age

The longevity of the art industry in the city where the university is located may affect the satisfaction of art-related programs. We use “Firm age” as a control variable, which is calculated based on the average age of enterprises in that city (data comes from “Tianyancha”, see “data.xlsx” and “National Enterprise Information (Chinese Version).xlsx” in the Supplementary files, and Table S1 in Appendix Sect. 2.1).

##### Business diversity

The business diversity of art industry-related enterprises in different cities may be related to the maturity of the industry and may have an exogenous impact on satisfaction. “Business diversity” is calculated based on the average length of text content related to business operations in the preliminary registration data from the local Industrial and Commercial Bureau (data comes from “Tianyancha”, see “data.xlsx” and “National Enterprise Information (Chinese Version).xlsx” in the Supplementary files, and Table S1 in Appendix Sect. 2.1).

##### Capital size

The capital size of art industry-related enterprises in different cities may also have an exogenous impact on satisfaction and is considered a control variable. “Capital size” is calculated as the total registered capital (in Chinese yuan) of related enterprises in each city (data comes from “Tianyancha”, see “data.xlsx” and “National Enterprise Information (Chinese Version).xlsx” in the Supplementary files, and Table S1 in Appendix Sect. 2.1).

##### Number of votes

The satisfaction information provided by CHSI includes the cumulative number of votes for each program. This number reflects the scale of students in the program. Therefore, using the number of votes as a control variable to observe satisfaction is reasonable (see “data.xlsx” in the Supplementary files).

##### Female-male ratio

According to the consulting information provided by the Yifan Career Planning System, the gender distribution of each program is shown in Table 1. The information held by this institution is based on professional data collection and consulting industry experience, ensuring not only relatively reliable sources of information but also maintaining consistent statistical standards. Similarly to the variable of gender ratio, although the gender distribution within the same program may vary across different institutions and different times, the average trend remains similar. This is due to the uniformity of the educational system and the open qualification criteria, thereby allowing the information in Table 1 to serve as a unified reference standard for the gender distribution of various programs.

A detailed explanation of the data sources, evaluation procedures, and reliability assessments for the control variables is provided in Appendix Sect. 2.6.

#### Dummy variables

Considering the potential heterogeneity among different arts disciplines, a simple classification method can be used to observe this possible phenomenon. To this end, we introduce a set of dummy variables that categorize 23 disciplines into 12 categories as 12 dummy variables. This approach allows for a more intuitive examination of whether there are significant differences in satisfaction levels across different types of disciplines. A detailed explanation of the dummy variables is provided in Table S4 in Appendix Sect. 2.8 and Tables S5 to S8 and Figure S1 in Appendix Sect. 4.1.

### Model

This study employs multiple regression analysis to examine the effects of content complexity and auditory-visual ratio on student satisfaction in arts-related programs. A sequential regression strategy is adopted, beginning with the inclusion of control variables, followed by the two focal independent variables, and finally incorporating their interaction term. The full regression model is specified as follows:

1 $$\begin{array}{c}{y}_{i}={\beta }_{0}+\sum_{m=1}^{p}{\gamma }_{m}{\cdot C}_{mi}+{\beta }_{1}\cdot {Content}_{i}+{\beta }_{2}{\cdot Avr}_{i}+{{\beta }_{12}\cdot Content}_{i}\times {Avr}_{i}+{\epsilon }_{i}\end{array}$$

where ${y}_{i}$ denotes the average satisfaction score for program $i$, and ${\beta }_{0}$ is the intercept. The variables ${Content}_{i}$ and ${Avr}_{i}$ represent the two independent variables: content complexity and auditory-visual ratio, respectively. The term ${C}_{mi}$ refers to the m-th control variable, with its associated coefficient ${\gamma }_{m}$. The interaction term ${Content}_{i}\times {Avr}_{i}$ captures the joint effect of the two focal predictors, with ${\beta }_{12}$ representing its coefficient. The error term ${\epsilon }_{i}$ accounts for unobserved variance at the program level and is assumed to be normally distributed.

## Results

Table 2 presents the summary statistics, and Table 3 shows the correlation matrix, including the dependent variable, three independent variables, and control variables. All pairwise correlations are below 40%, meeting the independence requirements for multivariate regression analysis. The variance inflation factor (VIF) results are detailed in Appendix Sect. 4.1.

**Table 2:** Summary statistics of program-level variables.

Variable	Mean	Std. dev	Min	Max
Program satisfaction	4.690	0.218	3.400	5.000
Beijing	0.045	0.208	0.000	1.000
Tianjin	0.024	0.152	0.000	1.000
Shanghai	0.024	0.154	0.000	1.000
Chongqing	0.024	0.152	0.000	1.000
Department level	1.027	0.385	0.000	2.000
Firm age (log)	2.224	0.259	0.264	3.490
Business diversity (log)	5.504	0.267	4.290	6.865
Capital size (log)	10.577	3.298	− 1.308	15.118
F/M ratio (log)	0.704	0.297	− 0.040	1.266
Voters (log)	3.475	0.904	1.609	6.157
Content complexity (log)	2.668	0.349	1.609	2.996
A/V ratio (log)	− 0.234	0.530	− 1.253	0.470

N = 1524.

A/V ratio: Auditory_visual ratio.

F/M ratio: Female_male ratio.

**Table 3:** Correlation matrix and variance inflation factors (VIF) for all variables.

ID	Variables	1	2	3	4	5	6	7	8	9	10	11	12	13
1	Program satisfaction	1												
2	Beijing	0.071**	1											
3	Tianjin	0.109***	− 0.034	1										
4	Shanghai	− 0.012	− 0.034	− 0.025	1									
5	Chongqing	0.041	− 0.034	− 0.024	− 0.025	1								
6	Department level	0.041	0.059*	0.382***	0.222***	0.281***	1							
7	Firm age	− 0.004	0.122***	− 0.037	0.043	0.046	0.015	1						
8	Business diversity	− 0.023	0.079**	− 0.015	− 0.029	0.108***	− 0.016	− 0.121***	1					
9	Capital size	0.086***	0.300***	− 0.009	0.172***	0.043	0.024	0.183***	0.288***	1				
10	female_male ratio	− 0.072**	− 0.096***	− 0.045	− 0.046	− 0.035	− 0.038	− 0.023	− 0.009	− 0.217***	1			
11	voters	− 0.383***	− 0.057*	− 0.102***	− 0.030	− 0.051*	− 0.049	0.023	0.010	− 0.068**	0.125***	1		
12	Content complexity	− 0.064*	− 0.100***	− 0.011	− 0.037	0.007	− 0.013	− 0.046	− 0.047	− 0.070**	− 0.128***	− 0.026	1	
13	Auditory_visual ratio	− 0.054*	− 0.010	− 0.043	− 0.026	− 0.005	− 0.017	− 0.017	− 0.000	− 0.087***	0.221***	0.131***	− 0.101***	1

N = 1524.

*** *p* < .001; ** *p* < .01; * *p* < .05; † *p* < .1.

Table 4 displays the direct effects of the two independent variables on student satisfaction. Model 1 represents the regression effects with control variables. Models 2 and 3 analyze the effects of content complexity and A/V ratio on satisfaction, respectively. Model 4 includes both independent variables in the statistical test as main effects. From Model 2 and Model 4, it can be observed that content complexity has a stable and significant negative effect on satisfaction (*p* < 0.01), providing strong support for H1 in the CLT framework.

**Table 4:** Direct effects of content complexity and A/V ratio on program satisfaction.

Variable	Model 1	Model 2	Model 3	Model 4
Constant	5.199***	5.342***	5.200***	5.342***
	(0.127)	(0.136)	(0.128)	(0.136)
***Control variables***				
Beijing	0.040	0.032	0.039	0.032
	(0.026)	(0.026)	(0.026)	(0.026)
Tianjin	0.110**	0.107**	0.110**	0.107**
	(0.038)	(0.038)	(0.038)	(0.038)
Shanghai	− 0.041	− 0.045	− 0.041	− 0.045
	(0.036)	(0.035)	(0.036)	(0.035)
Chongqing	0.045	0.045	0.045	0.045
	(0.036)	(0.036)	(0.036)	(0.036)
Department level	− 0.008	− 0.007	− 0.008	− 0.007
	(0.016)	(0.016)	(0.016)	(0.016)
Firm age	− 0.013	− 0.015	− 0.013	− 0.015
	(0.021)	(0.021)	(0.021)	(0.021)
Business diversity	− 0.039†	− 0.041*	− 0.039†	− 0.041*
	(0.021)	(0.021)	(0.021)	(0.021)
Capital size	0.005*	0.004*	0.005*	0.004*
	(0.002)	(0.002)	(0.002)	(0.002)
F/M ratio	− 0.004	− 0.012	− 0.005	− 0.012
	(0.018)	(0.018)	(0.018)	(0.018)
Voters	− 0.088***	− 0.089***	− 0.088***	− 0.089***
	(0.006)	(0.006)	(0.006)	(0.006)
***Independent variables***				
Content complexity		− 0.045**		− 0.045**
		(0.015)		(0.015)
A/V ratio			0.002	− 0.001
			(0.010)	(0.010)
F-Statistics	28.909***	27.240***	26.266***	24.954***
Adjusted R-Square	0.155	0.159	0.154	0.159
ΔAdjusted R-Square	–	2.88%	− 3.14%	2.88%
Degrees of Freedom	1513	1512	1512	1511

N = 1524.

The dependent variable (DV): Program satisfaction.

Standard errors in parentheses.

*** *p* < .001; ** *p* < .01; * *p* < .05; † *p* < .1

In contrast, the A/V ratio does not show significant effects in either Model 3 or Model 4 (*p* > 0.05), offering no direct evidence for H2 at this stage. All reported coefficients are partial regression coefficients, reflecting the unique contribution of each predictor net of other variables.

Table 5 further tests the hypothesized interaction effect. Model 2 introduces the interaction term between content complexity and A/V ratio. The coefficient for A/V ratio becomes significantly negative (β =  − 0.208, *p* < 0.05), and the interaction term is positive and significant (β = 0.077, *p* < 0.05), lending support to H2 and H3. The moderation effect suggests that visual emphasis may buffer the negative effect of content complexity on satisfaction. Meanwhile, content complexity remains a negative predictor (β =  − 0.043, *p* < 0.01), though its magnitude slightly diminishes in Model 3. A more detailed analysis of hierarchical regression, adjusted R^2^, dummy variables, and robustness tests is provided in Tables S5 to S10 in Appendix Sect. 4.

**Table 5:** Interaction effect of A/V ratio and content complexity on program satisfaction.

Variable	Model 1	Model 2	Model 3
Constant	5.199***	5.342***	5.337***
	(0.127)	(0.136)	(0.136)
***Control variables***	*Entered*	*Entered*	*Entered*
***Independent variables***			
Content complexity		− 0.045**	− 0.043**
		(0.015)	(0.015)
A/V ratio		− 0.001	− 0.208*
		(0.010)	(0.090)
(Content complexity) × (A/V ratio)			0.077*
			(0.033)
F-Statistics	28.909***	24.954***	23.508***
Adjusted R-Square	0.155	0.159	0.161
ΔAdjusted R-Square	–	2.52%	1.51%
Degrees of Freedom	1513	1511	1510

N = 1524.

The dependent variable (DV): Program satisfaction.

Standard errors in parentheses.

*** *p* < .001; ** *p* < .01; * *p* < .05; † *p* < .1.

## Discussion

This study interprets content complexity as a manifestation of instructional abstraction. Through the lens of CLT, increased complexity increases psychological distance, which in turn dampens emotional satisfaction—particularly in disciplines with concrete default environments such as the arts. We discuss the fundamental question of the relationship between the complexity of teaching content and student satisfaction within art disciplines. By linking content complexity with educational abstraction and employing the CLT framework, statistical analysis of voting data from 73,368 Chinese art students confirms that higher complexity in educational content significantly decreases student satisfaction. Clarifying this core issue not only addresses long-standing debates within this specific educational context but also provides insights into improving educational policies through the unique lens of CLT.

CLT naturally explains why, overall, increasing teaching content complexity leads to a notable decline in satisfaction. However, this conclusion primarily applies to art-related disciplines or fields with similar characteristics. For other types of disciplines, targeted data and evidence are still lacking. Based on CLT principles, it is plausible that disciplines inherently aligned with abstract thinking may yield findings contrary to those of this study. This heterogeneity underscores the necessity of focused research and reflects the broad applicability of CLT.

The "construal fit effect" emphasizes that aligning abstraction and concreteness levels is critical for satisfaction. Abstract issues benefit from emphasizing abstract elements, while concrete issues are better addressed with concrete features. This study categorizes student satisfaction into high-level abstraction and low-level concreteness, confirming that program satisfaction aligns with concreteness.

Key independent variables—content complexity and the auditory-visual (A/V) ratio—reflect the abstraction level of educational content and program modalities. Both variables significantly affect student satisfaction, as greater concreteness correlates with higher satisfaction. When education becomes more abstract, introducing additional abstract adaptation factors can alleviate dissatisfaction. A higher A/V ratio moderates the main effect, demonstrating that pairing abstract content with a higher A/V ratio increases satisfaction. Interaction effects reveal that while a higher A/V ratio directly reduces satisfaction, it enhances satisfaction when the content is abstract. This aligns with the CLT framework, where abstract educational content requires more abstract forms and modes of thinking.

This analysis addresses a longstanding and unresolved question within CLT: when the environmental characteristic (default psychological distance) and interaction characteristic (decision-making approach) are fixed and do not achieve fit, should supplementary decisions prioritize fitting with the environment or with existing decisions? Both approaches seem theoretically plausible under CLT. Through the verification of Hypothesis 3, this study clarifies that when faced with such a scenario, the abstraction level of supplementary decisions should align with that of the existing decisions, rather than the environment, to achieve better feedback outcomes.

The conclusions drawn from the verification of the three hypotheses in this study can be meaningfully extended beyond art-related fields and even outside education. The theoretical analysis and explanations provided adhere strictly to the core principles of CLT. However, when discussing areas such as marketing or decision-making, additional research is necessary to verify and explore potential heterogeneities to ensure the conclusions’ applicability. For instance, the process of categorizing abstraction levels in this study may be specific to its sample and context. Similarly, the fit pattern observed in Hypothesis 3 may depend on unobserved conditions, such as the influence of varying decision intensities or the sequential relationship between multiple decisions on the ultimate feedback direction. These uncertainties, while potentially impactful, require further clarification in subsequent research.

For now, this study offers a preliminary explanation of a typical and commonly encountered issue, laying the groundwork for future research. The findings serve as critical references for the next steps in advancing theoretical understanding and practical applications.

While this study focuses on student satisfaction rather than learning outcomes such as creativity or skill acquisition, its relevance extends beyond marketing or institutional branding. Satisfaction functions as a key indicator of students’ psychological engagement, emotional resonance, and perceived value—all of which are vital to sustaining learning motivation and long-term participation in arts education. By identifying structural mismatches between educational content and student expectations, the findings provide practical implications for curriculum designers, university administrators, and policymakers aiming to improve retention, inclusiveness, and responsiveness in art programs. Moreover, the results contribute theoretically by extending CLT to an underexplored domain—arts education—and by clarifying how abstract-concrete dynamics interact in shaping affective feedback under complex cognitive environments.

One potential concern from readers may be whether satisfaction feedback might be influenced by employment outcomes, potentially confounding the study’s conclusions, or the issue of voter distribution bias. We discuss this potential problem in detail in Appendix and conclude that there is no need to worry about these potential endogeneity factors. See Appendix Sect. 4.3 for details.

Additionally, we recommend that the A/V ratio be interpreted as an auxiliary descriptor of macro-level perceptual structure, rather than as a direct proxy for instructional design. It primarily serves to signal perceptual modality preferences at the disciplinary level, helping to contextualize students’ psychological responses (see Appendix Sect. 5 for further discussion of limitations and implications).

## Conclusion

This study makes three key theoretical contributions. First, it explores the relationship between the educational characteristics of arts-related programs and student satisfaction, based on data from 1,524 independent programs across China. While previous research has primarily focused on perceived value, perceived quality, ability traits, and personality as determinants of satisfaction, this study shifts attention to educational factors, applying the lens of psychological distance and addressing heterogeneity across artistic disciplines.

Second, by incorporating Construal Level Theory (CLT), the study extends its application to the domain of arts education, offering a novel perspective for interpreting how abstraction affects satisfaction outcomes.

Third, it systematically examines both the direct and moderating effects of abstraction-related variables—namely, content complexity and modality structure (A/V ratio)—on satisfaction. Through an integrated framework of theoretical reasoning and empirical testing, the study deepens the explanatory utility of CLT in educational research.

In practice, this study offers actionable insights for improving the educational system in arts-related disciplines. The economic and innovative development of society is closely tied to the prosperity of the arts industry, which, in turn, relies on the satisfaction of arts students. This study proposes two strategies to enhance the educational experience in these fields.

1. Reducing abstraction when possible. Institutions can enhance satisfaction by lowering content complexity through greater interactivity, intuitive course materials, and stronger links between coursework and real-world application. In programs that prioritize experience over rigor, incorporating pleasure-oriented, hands-on approaches may yield more emotionally resonant and satisfying outcomes by reducing psychological distance and reinforcing concreteness.
2. Achieving abstraction fit when complexity is inevitable. In cases where high abstraction is necessary to maintain academic depth, it is crucial to match this abstraction with similarly abstract instructional strategies to avoid cognitive dissonance. In this study, the auditory-visual ratio serves as a proxy for such strategies, though the underlying principle can extend to communication methods, instructional formats, cognitive framing, and assignment design. Additionally, fostering future-oriented reflection among students may also align well with abstract educational environments under the CLT framework.

While the findings are drawn from arts-related disciplines, the underlying mechanisms identified—particularly those concerning construal fit—may have broader applicability across fields where perceptual modes and abstraction levels interact.

One potential limitation of this study is that it does not directly control for students’ academic ability, which may be associated with both program content complexity and reported satisfaction. Although the current analysis includes several contextual controls—such as institutional hierarchy and city-level economic indicators—future research could further improve model precision by incorporating academic ability proxies, such as university entrance scores or program-level GPA averages, if available.

## Electronic supplementary material

Below is the link to the electronic supplementary material.

Supplementary Material 1

Supplementary Material 2

Supplementary Material 3

## Data Availability

All data generated or analyzed during this study are included in this published article (and its Supplementary Information files).
