# Supplementary material for: How content complexity and sensory modality influence student satisfaction in art education
Source: Sci Rep. 2025 Jul 1;15:21156. doi: 10.1038/s41598-025-08242-5 (PMC12219321; doi:10.1038/s41598-025-08242-5)
Supplement: Supplementary file 1 — Supplementary Material 1 [file 41598_2025_8242_MOESM1_ESM.pdf]

Appendix

Table of Contents

Appendix 1

1. Why Construal Level Theory is adopted 2

1.1. Why CLT is an appropriate theoretical framework (Revised) 2

1.2 Why Other Theoretical Frameworks Were Not Selected 4

2. Data Sources and Variable Construction 5

2.1 Data Sources 5

2.2 Clarification of Terminology: Disciplines, Programs, and Units of Analysis 6

2.3 Data Handling and Analytical Methods 7

2.4 Selection and Operationalization of the Independent Variable: Content Complexity 7

Data Collection and Evaluation Procedure 8

Quantification of Content Complexity 8

Objectivity and Reliability of the Evaluation 9

2.5 Selection and measurement of the auditory-visual ratio 10

Measurement procedure 11

Expert validation and scoring 11

Overview of results 12

2.6 Selection and measurement of control variables 12

2.6.1 Municipality status 13

2.6.2 Department level 13

2.6.3 Firm age 13

2.6.4 Business diversity 13

2.6.5 Capital size 14

2.6.6 Female-male ratio 14

2.6.7 Number of votes 14

2.7 Reliability of expert evaluations 14

2.8 Dummy variables 15

3. Data processing 16

3.1 Data handling procedures 16

3.2 Logarithmic transformation 16

4. Extended analysis of the model 17

4.1 Dummy variable analysis 17

Preliminary analysis: mean differences across disciplines 17
Regression analysis including discipline dummies 18
4.2. Hierarchical Linear Model 22
Interpretation 23
4.3. Addressing Potential Endogeneity Concerns 23
4.4 Model Effect Analysis 25
4.5. On the Explanatory Power of the Model 26
5. Positioning and Limitations of This Study 27
5.1 Theoretical positioning: Student satisfaction in the context of art education 27
5.2 Interpretive boundaries and limitations 28
5.3 Implications for Future Research 30
6. Ethics statement 30
Student satisfaction data (dependent variable): 30
Course data (independent variable): 30
Enterprise information data (control variable): 30
University official websites data: 31
Public information from Baidu search: 31
Expert interviews: 31

1. Why Construal Level Theory is adopted

1.1. Why CLT is an appropriate theoretical framework (Revised)

Construal Level Theory (CLT), developed by Trope and Liberman (2010), proposes that people interpret objects, events, and information at different levels of mental abstraction based on their perceived psychological distance. This distance can be temporal (how far in the future or past), spatial (how physically distant), social (how dissimilar or detached the entity is), or hypothetical (how likely or unlikely an event is). Events that are psychologically distant tend to be processed using high-level, abstract representations (e.g., general concepts and goals), while psychologically near events are understood in more concrete, contextualized terms (e.g., specific actions and experiences).

CLT has been widely applied in studies on consumer judgment, decision-making, and message framing. In recent years, it has gained traction in educational research and affective evaluation because it offers a psychologically grounded explanation for how individuals process complex or abstract information. In the context of this study, educational content complexity and media modality can be viewed as key drivers of psychological distance. More abstract or conceptually complex courses may increase perceived distance

between the student and the learning material, potentially leading to lower satisfaction. In contrast, rich visual elements or more concrete content structures may reduce psychological distance, thus enhancing evaluative judgments.

Importantly, CLT also emphasizes the construal fit mechanism, which suggests that individuals respond more positively when the level of abstraction in the content matches the psychological distance of the context. In other words, abstract content is more effective when the context is also perceived as abstract, and concrete content works better in concrete settings. This fit between content and context enhances cognitive fluency and evaluative coherence, often leading to greater satisfaction.

This notion of construal fit is particularly relevant in art education, where increasing curriculum complexity and conceptual abstraction may not align with students' perceived proximity to their programs. When abstraction is unavoidable—such as in advanced or interdisciplinary art programs—adjusting the modality or delivery format (e.g., increasing abstract features such as auditory emphasis) can help reestablish fit and mitigate dissatisfaction. This conceptual alignment provides a compelling rationale for using CLT not only to explain direct effects of abstraction, but also to theorize and empirically test interaction effects—a central focus of this study.

Recent empirical research increasingly affirms the relevance of Construal Level Theory (CLT) in the study of satisfaction and well-being. CLT distinguishes between high-level (abstract) and low-level (concrete) construals, offering a nuanced lens through which individuals' evaluative judgments can be understood. This theoretical framework helps explain how people process, internalize, and respond to life experiences across various psychological distances.

On the one hand, high-level construals have been associated with enhanced long-term satisfaction. For instance, (Alfalah & Alganem, 2020) found that individuals inclined toward abstract thinking reported greater happiness, optimism, and overall life satisfaction—likely because abstract processing encourages broader, goal-oriented evaluations. Similarly, (Hu et al., 2023) demonstrated that high-level construals mediate the relationship between cognitive focus and financial satisfaction, helping reduce emotional ambivalence and enabling more stable self-assessments. (Sun et al., 2021) further noted that abstract construals can buffer the negative emotional effects of internal conflict, reinforcing their value in supporting psychological resilience.

On the other hand, recent findings also highlight the benefits of low-level construals, particularly in contexts that demand attentiveness to immediate circumstances. (Crouzevialle et al., 2023) showed that concrete, detail-focused thinking can increase short-term satisfaction by heightening awareness of positive experiences and reducing stress. (Caballero et al., 2024) similarly found that in situations such as financial

scarcity, low-level construals help individuals focus on attainable, near-term benefits, thereby enhancing subjective well-being.

Taken together, these studies suggest that both abstract and concrete construals play adaptive roles in shaping satisfaction, depending on context and individual needs. While abstract thinking facilitates long-term meaning-making and emotional regulation, concrete thinking supports responsiveness to present conditions and emotional immediacy. An integrative view of CLT is thus emerging—one that embraces the dynamic interplay between construal levels as a foundation for understanding satisfaction and well-being in complex environments.

1.2 Why Other Theoretical Frameworks Were Not Selected

While several well-established psychological theories could potentially offer insights into student satisfaction, they do not align as directly with the specific constructs and mechanisms under investigation in this study as Construal Level Theory (CLT) does. Our central interest lies in how intrinsic attributes of course design—namely content complexity and sensory modality—shape student satisfaction through cognitive-perceptual processing. This research focus calls for a framework that explains how individuals mentally construe structural aspects of information and how these construals influence affective evaluations.

Social Identity Theory (SIT) (Brown, 2000; Stets & Burke, 2000), for example, has been widely used to explain how affiliation with social groups shapes self-concept, belonging, and well-being. Studies such as Petrakis and (Petrakis & Kanzola, 2022) demonstrate the utility of SIT in understanding satisfaction within the creative economy, particularly in contexts involving identity formation and group-level dynamics. However, the present study does not examine intergroup comparisons, identity salience, or institutional belonging. Rather, it focuses on how students internally process the design characteristics of their academic programs. In this context, SIT offers less explanatory precision than CLT for our particular research objectives.

Similarly, Expectancy-Value Theory and Self-Determination Theory offer important perspectives on motivation and learning outcomes, emphasizing factors such as perceived value, competence, and autonomy. These frameworks are highly relevant to understanding why students engage with academic content. However, our study is concerned with how the structural features of that content—particularly its abstractness and sensory configuration—shape satisfaction through psychological distance. Unlike CLT, these motivational theories do not provide a cognitive mechanism to account for how abstraction and modality jointly influence evaluative judgments.

In contrast, CLT offers a unified and empirically testable account of how content complexity and modality interact through the lens of psychological distance. This makes it particularly well-suited for exploring both the main effects and moderating relationships in our research model. It is important to note

that the use of CLT in this study does not preclude the value of other theoretical perspectives. On the contrary, future research could build upon this framework by incorporating motivation- or identity-based moderators, drawing from theories like SIT or EVT to expand the explanatory scope.

2. Data Sources and Variable Construction

2.1 Data Sources

This study draws on four primary data sources, covering higher education indicators, urban economic conditions, gender distribution, and academic program characteristics.

The first source is the China Higher Education Student Information (CHSI) platform, an official system administered by the Student Service and Development Center under the Ministry of Education. The platform provides comprehensive data on higher education institutions, including program distribution, institutional scale, and satisfaction ratings. The satisfaction data are collected through a real-name registration voting system, targeted at undergraduate students approaching graduation. The survey consists of a single-item question: "How satisfied are you with your academic program?"

The dataset includes 693 universities across China, covering 23 disciplines related to the cultural and entertainment industries, with a total of 1,524 academic programs. The CHSI data used in this study were collected in April 2024 and represent cumulative responses from 2008 to 2024. The final sample comprises 73,368 undergraduate graduates from 198 cities across 31 provinces and municipalities. The data are publicly accessible and fully anonymized to ensure the exclusion of personally identifiable information, making them a reliable and ethically compliant foundation for empirical analysis.

The second source is Tianyancha, a commercial platform for enterprise information synchronized with data from China's official market regulation authorities. This study focuses on firms in the cultural and entertainment sectors. To be included in the dataset, enterprises must meet specific criteria: they must be domestically owned, currently active, have complete contact and trademark information, and employ at least ten registered staff members. After applying these filters, the final dataset includes 9,038 valid enterprise records across 289 cities, with registration years ranging from 1956 to 2024.

Gender distribution data are obtained from the Yifan Career Planning Network, a platform that compiles publicly available statistics on gender ratios in undergraduate programs across Chinese universities. The reliability of the data has been verified by domain experts. In line with established research conventions, this study assumes that gender composition is homogeneous across institutions offering the same academic program. This assumption is widely accepted and supported by empirical evidence (Wang & Ieong, 2019; Zha, 2009a, 2009b).

The website information of the above three data sources is shown in Table S1.

Table S1. Data source URL information

ID	Website name	Chinese name	Properties	Openness
1	CHSI	学信网	Administered by the Ministry of Education	Full disclosure
	Website address	https://gaokao.chsi.com.cn/zyk/pub/myd/schAppraisalTop.action		
2	Yifan career planning system	一帆生涯	Private enterprise	Partial disclosure
	Website address	https://www.yifzy.com		
3	Tianyancha	天眼查	Private enterprise	Partial disclosure
	Website address	https://www.tianyancha.com		

Finally, due to the lack of publicly available data on the structural knowledge components of academic curricula, the assessment of these features was conducted manually based on the proportion of abstract versus concrete elements. The evaluation drew on multiple data sources, including course syllabi from the Yifan Career Planning System, resources published by the Ministry of Education, official university websites, and third-party higher education consulting platforms. In addition, expert feedback was obtained from six higher education professionals located in diverse regions across China, including Shenyang, Changchun, Guilin, Guangzhou, Beijing, and Luoyang. This multi-source approach ensured the completeness and consistency of the data, providing a robust foundation for subsequent analyses.

2.2 Clarification of Terminology: Disciplines, Programs, and Units of Analysis

To ensure conceptual clarity for international readers, this section defines the hierarchical structure of academic categories and sample units used in this study. The classification is based on the official framework of Chinese higher education and is organized into multiple levels:

(1) **First-level disciplines** refer to broad academic domains formally recognized in national education standards. In this study, we categorize the data into 12 first-level disciplines, which are used to construct dummy variables in the regression models (e.g., Music Studies, Fine Arts, Design and Media, Performing Arts).

(2) **Second-level disciplines** represent more specific academic fields within each first-level discipline, such as Music Education, Product Design, or Broadcast and Hosting Art. The dataset includes 23 second-level disciplines, which serve as the primary classification of subject areas for satisfaction reporting.

(3) **Programs** refer to distinct academic offerings operated by individual universities, such as Music Education at Tsinghua University. Each program is uniquely identified by both its disciplinary designation and institutional affiliation. **The 1,524 programs analyzed in this study are directly expanded from the 23 second-level disciplines**—that is, each second-level discipline contains multiple university-level program instances across the country. These 1,524 programs constitute the main units of analysis. All variables, including satisfaction scores and program characteristics, are measured at this level.

(4) Student-level satisfaction responses were originally submitted by graduating undergraduates via the CHSI platform through a real-name voting system. The dataset contains 73,368 individual responses

collected between 2008 and 2024. However, the CHSI database only reports aggregated satisfaction scores at the **program level**, and does not release individual-level response data. Therefore, this study is necessarily conducted at the program level, with each of the 1,524 programs representing a single analytical unit based on its corresponding average satisfaction score.

(5) In this study, the dependent variable "program satisfaction" refers to students' aggregated satisfaction at the program level. While the construct itself represents student satisfaction, the term "program satisfaction" is used to emphasize the unit of analysis—namely, the academic program. Throughout the manuscript, the terms "program satisfaction" and "student satisfaction" may occasionally appear interchangeably; however, they refer to the same underlying concept, with the former highlighting the analytical level and the latter emphasizing the individual perspective.

This structure allows the study to model how program-level characteristics—such as content complexity and audiovisual modality—influence student satisfaction, while also accounting for discipline-level categorization and broader institutional variation. The 1,524 programs analyzed in this study are specific instances derived from the 23 second-level disciplines, each representing a distinct institutional implementation.

2.3 Data Handling and Analytical Methods

The data acquisition process combined both automated and manual methods. For CHSI data, we used a hybrid approach involving Python scripts for extraction and manual validation. For Tianyancha, enterprise data were accessed and downloaded through a paid institutional membership. Gender ratio data from the Yifan Career Planning Network were manually collected using a registered account with access to publicly available information. Additional third-party information was retrieved manually from publicly accessible web-based sources. All data collection procedures adhered to relevant internet usage regulations and did not involve any personally identifiable information.

Before conducting regression analysis, the datasets were organized and formatted to ensure consistency across sources and to prepare them for statistical analysis. Excel was used as the primary data management platform, while MATLAB (version 2021a) was employed for variable construction and transformation. Tasks performed in MATLAB included the quantification of control variables, calculation of independent variables, logarithmic transformations, and other computation processes relevant to this study.

For statistical analysis, the study primarily employed standard multiple linear regression models to test the proposed hypotheses. Most of the regression analyses were conducted using Stata (version 17.0). Visualizations and summary tables were generated using a combination of MATLAB (2021a) and Stata.

2.4 Selection and Operationalization of the Independent Variable: Content Complexity

The variable Content Complexity is selected as a core explanatory variable in this study because it captures the inherent cognitive and structural characteristics of academic programs that are highly relevant to students' evaluative experiences. In the context of higher education—particularly within arts and design disciplines—the degree to which course content is abstract, interdisciplinary, or conceptually demanding can shape students' engagement, perceived difficulty, and emotional response.

From a theoretical perspective, Construal Level Theory (CLT) offers a direct explanation for how such complexity may affect satisfaction. According to CLT, individuals interpret abstract or psychologically distant information using high-level mental construals, which tend to be less emotionally engaging and less personally relevant. In educational settings, highly complex content may increase psychological distance, making students perceive their learning experience as more detached, generalized, or less applicable to their immediate context—ultimately reducing satisfaction. Conversely, concrete and easily digestible content may foster psychological proximity and improve affective engagement.

Although prior studies have rarely operationalized abstraction or complexity at the program level, this study introduces Content Complexity as a theoretically grounded and methodologically novel construct to examine the affective impact of curriculum structure.

Data Collection and Evaluation Procedure

Content Complexity is defined as the degree of conceptual and categorical complexity embedded in the curriculum of each academic discipline. Two dimensions were considered for quantification:

(1) Number of courses: We used curriculum data from the "Yifan Career Planning System," a professional educational consulting service, as the primary reference. The number of courses in the standard curriculum for each of the 23 arts-related disciplines was extracted. These course lists were then cross-checked and corrected based on publicly available data from official university websites and the Ministry of Education to ensure consistency and reliability.

(2) Scale of course categories: In practice, many universities assign different names to similar or extended versions of the same course, which may artificially inflate course counts. To address this, we performed a manual categorization process to merge semantically overlapping or redundant course names into unified categories. This process was carried out in consultation with six experienced arts education experts from universities across various regions of China (including Shenyang, Changchun, Guilin, Guangzhou, Beijing, and Luoyang). This manual review ensured that the number of distinct course categories reflected the true curricular complexity of each discipline.

All data were compiled and reviewed between February and April 2024.

Quantification of Content Complexity

Based on the two-stage evaluation process described above, we constructed a numerical complexity score for each of the 23 disciplines. For example, Music Performance has a content complexity score of 19, indicating that—after standardization of course categories—it includes 19 distinct, standard courses that are commonly offered across institutions nationwide. This level of consistency is made possible by the highly standardized nature of China's national education system, where curriculum designs are subject to centralized regulation, and the variation in instructional hours for similar courses remains relatively limited. These institutional features justify the use of course count as a reliable proxy for program-level content volume and complexity.

To normalize the distribution of the complexity variable and improve the robustness of regression estimates, the final variable was log-transformed. For instance, a discipline with a raw complexity score of 19 (e.g., Music Performance) yields a log-transformed value of 2.944 (see Table S2). A more detailed justification for the logarithmic transformation is provided in Appendix 3.

Table S2. Content complexity scores and log-transformed values for 23 arts-related disciplines

ID	Discipline	Content complexity	Content complexity (Log)
1	music performance	19	2.944
2	music	18	2.890
3	Theory of Composition and Composition Technology	10	2.303
4	musical education	8	2.079
5	Recording Art	10	2.303
6	Art Design	18	2.890
7	Visual Communication Design	20	2.996
8	product design	15	2.708
9	Clothing and Apparel Design	10	2.303
10	public art	11	2.398
11	Art and craft	16	2.773
12	Digital Media Arts	10	2.303
13	Art and Technology	20	2.996
14	Performance	5	1.609
15	Theatre	6	1.792
16	Filmology	12	2.485
17	Drama Film and Television Literature	19	2.944
18	Radio and television director	14	2.639
19	Theatrical and film director	8	2.079
20	Theatrical film and television art design	19	2.944
21	The art of broadcasting and hosting	16	2.773
22	animation	14	2.639
23	Photography and Production	14	2.639

Objectivity and Reliability of the Evaluation

Although the evaluation process involved a degree of human judgment, the subjectivity involved was minimal and tightly constrained. The primary input—the number of courses—was derived from publicly accessible and verifiable sources. Human involvement was limited to the standardization and merging of overlapping course titles, a process that does not require specialized interpretation. To further ensure reliability, the proposed classifications were independently reviewed by six arts education experts. Following two rounds of consultation and anonymous voting, all final scores (see Table S2) were approved by unanimous consensus.

2.5 Selection and measurement of the auditory-visual ratio

The auditory-visual (A/V) ratio is included as a key explanatory variable to capture the modality structure of educational content—namely, the relative emphasis placed on auditory-based (e.g., lectures, spoken explanations, reading text) versus visual-based (e.g., demonstrations, images, videos) instructional components. This variable reflects not only pedagogical format but also students' perceptual access to information, which may influence how they cognitively and emotionally engage with the course material.

From the perspective of Construal Level Theory (CLT), modality plays an important role in determining psychological distance. Visual content tends to be more concrete, perceptually rich, and spatially immediate, which reduces psychological distance and fosters stronger emotional engagement. In contrast, auditory or text-based content is more abstract, sequential, and symbolically mediated, which may increase psychological distance and reduce affective response. Including the A/V ratio allows us to examine how modality interacts with content complexity in shaping student satisfaction—particularly in arts-related disciplines, where sensory engagement is a core part of the learning experience.

Compared to content complexity, the assessment of the A/V ratio relies more heavily on expert judgment. However, this approach is particularly appropriate for the 23 arts-related disciplines examined in this study, where both visual and auditory modalities are central to the curriculum. The nature of these disciplines makes it feasible to systematically assess and quantify the intensity of each modality, enabling the construction of a standardized A/V ratio for each discipline.

During the evaluation process, we found that assessing the modality characteristics of these disciplines was less difficult than expected. For those familiar with the structure of higher education programs, it is often straightforward to identify the dominant sensory mode based on course content and learning objectives. For example, music education clearly places greater emphasis on auditory components, whereas visual communication design relies more heavily on visual processing. While this process is not entirely free from subjectivity, it was enhanced through cross-validation and expert consultation to improve objectivity and reliability.

Measurement procedure

In this study, the A/V ratio is defined as the relative strength of auditory and visual components embedded in a program's instructional content. To improve the precision of measurement, we developed a scoring framework based on both theoretical definitions and practical indicators:

(1) The visual intensity score (range: 1 to 10) reflects the degree to which a program relies on visual perception in its coursework and training activities. Reference criteria include: whether the curriculum is predominantly visual (e.g., painting, graphic design, film production), whether practical training emphasizes visual skill development, and whether the field's career paths demand strong visual communication (e.g., animation, fashion design).

(2) The auditory intensity score (range: 1 to 10) captures the reliance on auditory processing, evaluated based on whether the curriculum emphasizes listening-based skills (e.g., music composition, audio engineering), whether practice involves auditory tasks (e.g., performance, recording), and whether career outcomes are oriented toward auditory capabilities (e.g., broadcasting, music performance).

To conduct the evaluation, we collected course syllabi and training objectives for all 23 disciplines, and systematically analyzed the proportion of visual and auditory content. For example, music performance curricula typically include vocal and instrumental training, suggesting high auditory intensity; whereas digital media arts emphasizes visual design and motion graphics, indicating a higher visual load. In addition, we reviewed the practical training components of each program to cross-validate sensory demands—for instance, recording arts requires proficiency with sound equipment, whereas graphic design emphasizes spatial layout and aesthetic perception.

Expert validation and scoring

To ensure scoring reliability, we invited six higher education experts with disciplinary expertise in arts programs to independently evaluate each discipline's auditory and visual intensity. Experts rated both dimensions using a standardized rubric based on curriculum content and practice activities, with each dimension weighted at 50%. The final intensity scores for each modality were computed as the mean of expert ratings. Based on these scores, we calculated the A/V ratio using the following formula:

$$Auditory\_visual\ ratio = \frac{AcousticI\ ntensity\ Score}{Visual\ Intensity\ Score} \qquad (A1)$$

For example, music performance received an auditory score of 9 and a visual score of 7, yielding an A/V ratio of 1.286. In contrast, graphic design received an auditory score of 4 and a visual score of 9, yielding a ratio of 0.444.

Using this method, we evaluated the auditory-visual structure of all 23 disciplines. These discipline-level scores were then extended to all 1,524 program-level units according to their corresponding discipline.

Compared to gender ratio assumptions, such modality-based instructional consistency is more defensible, since programs offering the same discipline across institutions often follow similar teaching formats and knowledge structures in China's standardized education system.

Overview of results

Table S3 summarizes the expert-evaluated auditory and visual intensity scores for all 23 disciplines. For instance, music performance has an auditory intensity score of 9 and visual intensity score of 7, indicating strong auditory dominance; visual communication design has a visual score of 9 and auditory score of 4, reflecting high visual emphasis. These quantified results provide a robust basis for analyzing the effects of modality on student satisfaction within the broader regression framework.

Table S3. Auditory and visual intensity scores and ratio values for 23 arts-related disciplines

ID	Discipline	Auditory intensity	Visual intensity	Auditory / Visual	Auditory / Visual (Log)
1	music performance	9	7	1.286	0.251
2	music	8	6	1.333	0.288
3	Theory of Composition and Composition Technology	7	6	1.167	0.154
4	musical education	6	5	1.2	0.182
5	Recording Art	9	8	1.125	0.118
6	Art Design	5	9	0.556	-0.588
7	Visual Communication Design	4	9	0.444	-0.811
8	product design	3	8	0.375	-0.981
9	Clothing and Apparel Design	3	9	0.333	-1.099
10	public art	3	7	0.429	-0.847
11	Art and craft	2	7	0.286	-1.253
12	Digital Media Arts	6	8	0.75	-0.288
13	Art and Technology	5	8	0.625	-0.47
14	Performance	9	7	1.286	0.251
15	Theatre	7	6	1.167	0.154
16	Filmology	7	8	0.875	-0.134
17	Drama Film and Television Literature	6	7	0.857	-0.154
18	Radio and television director	6	7	0.857	-0.154
19	Theatrical and film director	7	8	0.875	-0.134
20	Theatrical film and television art design	4	9	0.444	-0.811
21	The art of broadcasting and hosting	8	5	1.6	0.47
22	animation	5	9	0.556	-0.588
23	Photography and Production	8	9	0.889	-0.118

2.6 Selection and measurement of control variables

This study includes a comprehensive set of control variables to account for regional, institutional, economic, and demographic factors that may influence student satisfaction independently of the program-level variables of interest. Specifically, the following control variables were incorporated:

(1) Municipality status (Beijing, Shanghai, Tianjin, Chongqing)

(2) Department level (central, provincial, or municipal governance)

(3) Firm age (average years since establishment of local art-related enterprises)

(4) Business diversity (average length of business scope descriptions)

(5) Capital size (total registered capital of creative enterprises)

(6) Female-male ratio (gender composition of students by discipline)

(7) Number of votes (proxy for program size and student participation rate)

Each control variable was selected based on theoretical relevance and empirical plausibility, and was measured using data from official education platforms, enterprise registration databases, and validated consulting sources. The subsections below provide detailed explanations of the rationale and operationalization for each control.

2.6.1 Municipality status

This set of dummy variables captures whether a program is located in any of China's four centrally governed municipalities—Beijing, Shanghai, Tianjin, or Chongqing. Each of these cities is represented by an independent binary indicator (e.g., Beijing = 1 if the program is located in Beijing; 0 otherwise). These cities hold privileged administrative status and benefit disproportionately from central government funding, national cultural platforms, and institutional resources. As a result, universities situated in these municipalities often provide superior educational infrastructure, richer student experiences, and stronger linkages to elite cultural networks and job markets.

To control for such location-specific structural advantages, we include four separate dummy variables in the model. Regional location information is available through the CHSI dataset, allowing each municipality to be reliably identified and coded.

2.6.2 Department level

Chinese universities are administered by various levels of government, which differ in funding capacity, policy access, and reputational status. This variable captures the governance level of each institution (0 = centrally affiliated, 1 = provincially governed, 2 = municipally governed). Data were obtained from the Ministry of Education classification list and cross-verified through institutional websites. This variable helps control for structural governance-related variation in student satisfaction.

2.6.3 Firm age

This variable captures the average age of art-related enterprises in each city, as a proxy for industry maturity. Following Construal Level Theory, a well-established local creative economy may reduce students' psychological distance from future careers, enhancing perceived relevance and satisfaction. Firm-level data were obtained from Tianyancha, filtered for legitimate, active, Chinese-funded companies with at least 10 registered employees. Each firm's founding year was used to compute its age, and these were averaged at the city level. These city-level values were then matched to the 1,524 program samples based on city location.

2.6.4 Business diversity

Business diversity refers to the functional breadth of local art-related enterprises and is operationalized as the average number of Chinese characters in each company's business scope description. This reflects industry complexity and cross-sector integration. More diverse cities may provide richer environmental cues, reducing students' semantic psychological distance from practical applications. Enterprise data were obtained from Tianyancha, with business scope text length calculated for each firm. City-level averages were then assigned to programs based on geographic location.

2.6.5 Capital size

This variable reflects the financial scale of the city's art-related industry. It is calculated as the total registered capital (in RMB) of relevant enterprises in each city, based on Tianyancha registry data. Well-capitalized industries signal viability and opportunity, potentially reducing students' hypothetical psychological distance from career goals. Including this measure helps isolate program effects from broader economic conditions.

2.6.6 Female-male ratio

Discipline-level gender composition data were obtained from the Yifan Career Planning System, which compiles statistics from nationwide higher education sources. Given China's centralized admissions system and culturally embedded gender-field norms, gender ratios within disciplines tend to be consistent across universities. Following prior studies (Wang & Ieong, 2019; Zha, 2009a, 2009b), we assigned one gender ratio to each of the 23 disciplines. These data provide a robust foundation for assessing how gendered composition relates to student experience, while minimizing institution-level variation.

2.6.7 Number of votes

This variable represents the total number of satisfaction responses submitted for each program on the CHSI platform, serving as a proxy for educational scale. Larger programs may experience different satisfaction dynamics due to crowding, impersonality, or resource allocation. To correct for right-skewed distribution and outliers, vote counts were log-transformed before analysis. This variable allows the model to account for size-related structural effects that may otherwise confound program-level estimates.

2.7 Reliability of expert evaluations

This study engaged six experienced arts education experts from universities across different regions of China. Their diverse geographic backgrounds ensured a broad and balanced perspective. Expert judgment was involved in two key phases of variable construction: (1) the classification of content complexity and (2) the scoring of the auditory-visual (A/V) ratio. Given the nature of these tasks, we argue that this expert panel is both sufficient and reliable.

First, for content complexity, the primary input was objective and publicly available curricular information. Experts were consulted not to independently generate scores, but to consolidate semantically

overlapping courses within each discipline to improve the precision and consistency of the final classification. This distinguishes our approach from studies that rely purely on subjective judgment without external data anchoring.

Second, the scoring of the A/V ratio did not involve specialized or technical assessments. In fact, the authors themselves could have completed this task independently based on clear instructional content. Nevertheless, we involved expert input to further enhance transparency and reduce individual bias. The rating process was supported by a rubric and involved both theoretical and practical criteria.

Third, unlike many studies where individual expert scores are simply averaged, our study employed a three-round consensus-based approach. The final values were discussed and fully agreed upon by all six experts and the research team. This collaborative process ensured that the final scores were not just statistically averaged, but qualitatively validated and unanimously approved.

In sum, the use of expert judgment in this study was not a methodological necessity but rather a redundant safeguard to strengthen the objectivity of variable construction. The unanimous agreement among six diverse experts further supports the reliability of our quantification procedures.

2.8 Dummy variables

This study analyzes 23 discipline-level categories within the field of arts education. To explore whether inherent characteristics of different academic fields are systematically associated with variations in student satisfaction, we coded disciplinary type into categorical variables for use in regression analysis.

To maintain model parsimony and analytical clarity, the 23 second-level disciplines (e.g., music performance, product design) were consolidated into 12 first-level categories, following the official classification scheme issued by the Chinese Ministry of Education. These 12 categories were each operationalized as a dummy variable (1 = belongs to category; 0 = otherwise), as detailed in Table S4.

Table S4. Classification of 23 arts-related disciplines into 12 first-level categories for dummy variable construction

ID_1	ID_2	Category Name	Discipline name	Obs_1	Obs_2
1	1	Visual Arts and Design	Visual communication design	235	423
2			Clothing and apparel design	80	
3			Photography and production	18	
4			Animation	90	
5	2	Art design	Art design	20	20
6	3	Performance	Performance	50	50
7	4	Music performance	Music performance	192	192
8	5	Performing arts and film studies	Theatre	1	29
9			Theatrical and film director	9	
10			Theatrical film and television art design	15	
11			Filmology	4	
12	6	Music studies and composition	Musical education	125	136
13			Theory of composition and composition technology	11	
14	7	Media arts and entertainment studies	Digital media arts	84	133

15			Drama film and television literature	36	
16			Recording art	13	
17			Art and craft	12	
18	8	Fine arts and technology	Art and technology	5	25
19			Public art	8	
20	9	Music	Music	195	195
21	10	Radio and television director	Radio and television director	96	96
22	11	The art of broadcasting and hosting	The art of broadcasting and hosting	103	103
23	12	Product design	Product design	122	122

ID_1: Second-level discipline number

ID_2: First-level discipline number (Dummy variables)

It is important to note that the grouping of dummy variables is inherently aligned with the content and sensory characteristics of the disciplines—particularly those associated with auditory-visual modality. As such, introducing all 12 dummy variables as control variables alongside the main explanatory variables in the same regression model may lead to multicollinearity, potentially distorting coefficient estimates and undermining interpretability.

To address this issue, we do not include these dummy variables in the primary regression models. Instead, a separate analysis is presented in Appendix Section 4.1, where we examine the potential effects of field classification and carefully evaluate possible multicollinearity concerns. This cautious strategy ensures that the integrity of our primary estimates for content complexity and A/V ratio is preserved, while still providing insight into field-specific differences in satisfaction outcomes.

3. Data processing

3.1 Data handling procedures

Section 2 of the Appendix has provided detailed descriptions of the data sources and construction methods for each variable. In addition, we did not exclude any observations from the dataset based on outlier screening, since no significant outliers were identified in the distributional diagnostics after log transformation. Thus, all valid observations were retained to preserve the completeness of the sample.

3.2 Logarithmic transformation

The primary regression models in this study include two key independent variables (content complexity and A/V ratio), ten control variables, and one dependent variable (program satisfaction). Extended analyses also incorporate twelve dummy variables and one interaction term. With the exception of the dependent variable, all continuous variables underwent log transformation prior to analysis. This section provides a rationale for this decision.

First, several control variables—particularly those reflecting city-level economic features (e.g., firm age, business diversity, capital size)—exhibit highly skewed distributions with large value ranges. It is well established in empirical literature that such variables benefit from log transformation to correct right-skewness, reduce the influence of extreme values, and better conform to linearity assumptions. Log

transformation also facilitates the interpretation of coefficients in terms of marginal diminishing effects, which is particularly relevant in economic data.

Second, the dependent variable (program satisfaction) is based on a five-point Likert scale. Consistent with standard practice in social science research, Likert-scale dependent variables are typically not log-transformed unless the distribution exhibits strong skewness. In our case, the satisfaction scores are approximately normally distributed within the 1–5 range, and thus were retained in their original form for analysis.

Finally, regarding the independent variables (content complexity and A/V ratio), while the original value ranges are relatively narrow, we opted for log transformation due to its potential benefits in improving model fit, reducing residual skewness, and enhancing interpretability of marginal effects. This treatment is aligned with empirical conventions in recent research, such as the approach adopted by (Park et al., 2024).

4. Extended analysis of the model

4.1 Dummy variable analysis

This study analyzes 23 discipline-level categories in arts-related higher education. To explore whether inherent characteristics of different academic fields may be associated with variations in student satisfaction, we categorized the 23 second-level disciplines into 12 first-level categories, following the official classification framework of the Chinese Ministry of Education.

It is important to emphasize that this categorization is highly aligned with the disciplinary characteristics of each field—particularly those involving auditory-visual modality—which may introduce multicollinearity if these dummies are included in the same regression model alongside the primary independent variables. To preserve the robustness and interpretability of the main model, we therefore analyze the dummy variables separately in this appendix section and treat them with appropriate caution.

Preliminary analysis: mean differences across disciplines

As a first step, we examined whether there is any systematic heterogeneity in satisfaction scores across the 12 discipline categories. Table S5 reports the results of independent-sample t-tests comparing the mean satisfaction levels across disciplines. Figure S1 provides a visual summary of these findings.

Table S5. Mean satisfaction scores and confidence intervals across 12 arts-related discipline categories

ID	Var name	Obs	Mean	Std.err.	Std.dev.	[95%conf.interval]	
D1	Visual Arts and Design	423	4.698	0.011	0.217	4.677	4.719
D2	Art design	20	4.655	0.038	0.170	4.575	4.735
D3	Performance	50	4.750	0.025	0.175	4.700	4.800
D4	Music performance	192	4.667	0.018	0.251	4.631	4.703
D5	Performing arts and film studies	29	4.745	0.033	0.178	4.677	4.813
D6	Music studies and composition	136	4.652	0.016	0.184	4.621	4.683
D7	Media arts and entertainment studies	133	4.747	0.017	0.198	4.713	4.781

D8	Fine arts and technology	25	4.696	0.042	0.209	4.610	4.782
D9	Music	195	4.661	0.015	0.213	4.630	4.691
D10	Radio and television director	96	4.680	0.024	0.234	4.633	4.728
D11	The art of broadcasting and hosting	103	4.676	0.024	0.244	4.628	4.723
D12	Product design	122	4.709	0.019	0.208	4.672	4.746

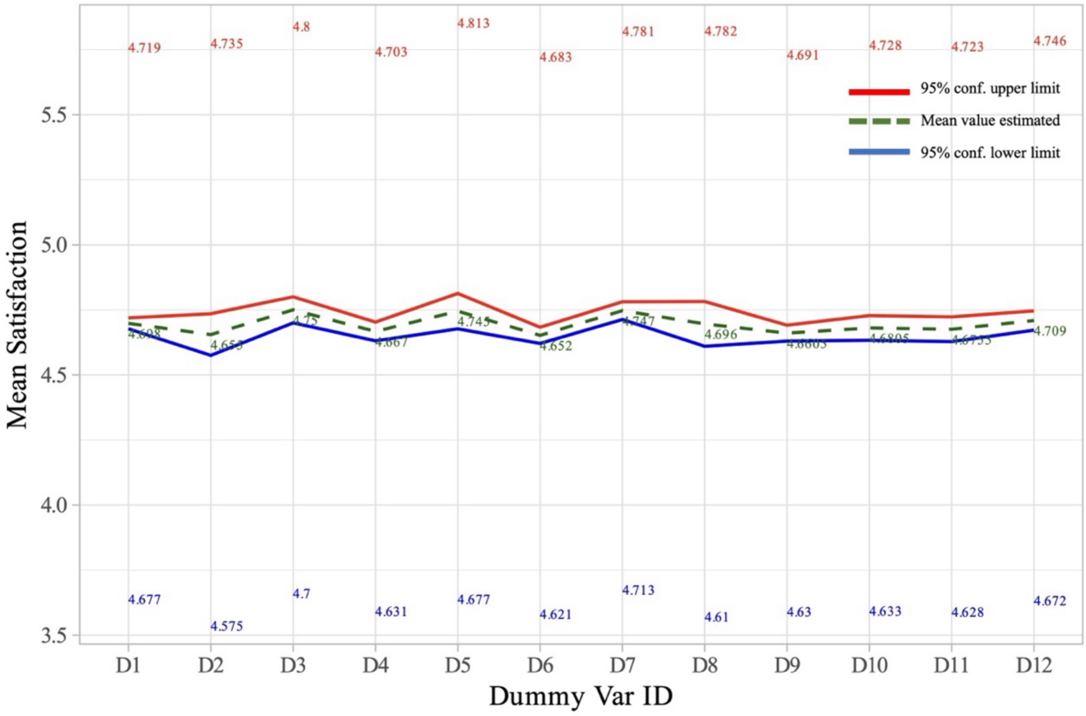

Figure S1. Mean satisfaction scores and confidence intervals across 12 arts-related discipline categories

As seen in Figure S1, although some variation exists in the mean satisfaction levels between disciplines, the differences are relatively small and do not exhibit any substantial or systematic disparities. This suggests that field-level differences in satisfaction are unlikely to drive discipline distortions in the main results and may not constitute a significant source of endogeneity.

Regression analysis including discipline dummies

To further investigate the relationship between discipline category and student satisfaction, we conducted an extended regression model that includes the following variables:

(1) The full set of 10 control variables

(2) 11 discipline-related dummies (with one category [D1] omitted as the reference group)

(3) The two key independent variables (content complexity and A/V ratio)

(4) Their interaction term

The regression results are presented in Table S6.

Table S6. Extended regression results including discipline dummies and main explanatory variables

Variable	Model 1	Model 2	Model 3	Model 4	Model 5

_cons	5.188***	5.372***	5.204***	5.392***	4.831***
	(0.129)	(0.154)	(0.131)	(0.157)	(0.221)
Control variables	*Entered*	*Entered*	*Entered*	*Entered*	*Entered*
Dummy variables					
D2_Art Design	-0.017	-0.010	-0.021	-0.015	-0.028
	(0.046)	(0.046)	(0.047)	(0.047)	(0.047)
D3_Performance	0.048	-0.026	0.018	-0.061	0.251*
	(0.031)	(0.046)	(0.059)	(0.069)	(0.111)
D4_music performance	-0.022	-0.011	-0.055	-0.048	-0.137*
	(0.017)	(0.018)	(0.057)	(0.057)	(0.062)
D5_Performing Arts and Film Studies	0.055	0.043	0.046	0.033	0.083†
	(0.039)	(0.039)	(0.042)	(0.042)	(0.044)
D6_Music Studies and Composition	0.013	-0.027	-0.025	-0.070	0.114
	(0.023)	(0.029)	(0.067)	(0.070)	(0.086)
D7_Media Arts and Entertainment Studies	0.051*	0.032	0.034	0.013	0.032
	(0.020)	(0.022)	(0.034)	(0.036)	(0.036)
D8_Fine Arts and Technology	0.007	0.001	0.014	0.009	0.006
	(0.041)	(0.041)	(0.043)	(0.043)	(0.043)
D9_music	0.023	0.032	-0.017	-0.012	-0.067
	(0.019)	(0.020)	(0.068)	(0.068)	(0.069)
D10_Radio and television director	0.008	0.000	-0.013	-0.023	-0.017
	(0.023)	(0.023)	(0.042)	(0.042)	(0.042)
D11_The art of broadcasting and hosting	-0.011	-0.011	-0.052	-0.056	-0.096
	(0.022)	(0.022)	(0.071)	(0.071)	(0.072)
D12_product design	0.009	0.003	0.019	0.014	0.006
	(0.021)	(0.022)	(0.027)	(0.027)	(0.027)
Independent variables					
Content complexity		-0.063*		-0.064*	0.158*
		(0.029)		(0.029)	(0.068)
A/V ratio			0.032	0.036	-0.852***
			(0.053)	(0.053)	(0.253)
(Content complexity)×(A/V ratio)					0.334***
					(0.093)
F-Statistics	14.590***	14.178***	13.937***	13.576***	13.647***
Adjusted R-Square	0.158	0.160	0.157	0.160	0.166
Δ Adjusted R-Square	-	1.33%	-1.54%	1.37%	4.11%
Degrees of Freedom	1502	1501	1501	1500	1499

N= 1524;
The dependent variable (DV): Program satisfaction;
Standard errors in parentheses;
*** p<.001; ** p<.01; * p<.05; † p<.1.

As shown in Table S6, the coefficients for the 11 dummy variables are generally small in magnitude and statistically non-significant, indicating that there is no strong association between field category and student satisfaction once other factors are controlled. This further supports the earlier finding that satisfaction scores do not systematically vary by disciplinary category at the aggregated (first-level) level.

Taken together, these results suggest that field-level heterogeneity, although conceptually relevant, does not exert a strong or confounding influence on students' evaluative responses in this context. As such, we conclude that the inclusion of dummy variables, while informative, does not alter the primary inferences of the study.

Another important methodological consideration involves the high degree of multicollinearity between the discipline dummy variables and the main explanatory variable A/V ratio. This overlap arises because the classification of dummy variables is inherently based on discipline-specific sensory characteristics, which are also central to the A/V ratio measure. To avoid inflating multicollinearity and destabilizing coefficient estimates, we chose not to include the dummy variables in the primary models presented in the main text (Tables 4 and 5). Instead, we analyze their effects separately in this appendix section.

As shown in Table S6, the coefficients for Content complexity and A/V ratio fluctuate across models as additional variables are included. This instability is symptomatic of underlying multicollinearity, particularly between the A/V ratio and the dummy variables.

To verify this, we computed Variance Inflation Factors (VIF) for different model configurations. Table S7 presents these diagnostics:

(1) Model configuration 1 includes all control variables, the full set of 11 dummy variables, and the two main explanatory variables (matching Model 4 in Table S6). Under this specification, four dummy variables—D4, D6, D9, and D11—exhibit VIF values substantially greater than 10, indicating severe multicollinearity with A/V ratio.

(2) In Model configuration 2, these four high-VIF dummies were removed. The multicollinearity problem was significantly alleviated, with the maximum VIF reduced to 1.89, well below conventional diagnostic thresholds.

Table S7. Variance Inflation Factor (VIF) diagnostics for alternative model specifications

Variable	VIF		
Variable Combination	1	2	3
A/V ratio	30.16	1.37	1.07
D9_Music	19.54	*Omited*	*Omited*
D6_Music studies and composition	15.1	*Omited*	*Omited*
D4_Music performance	13.7	*Omited*	*Omited*
D11_The art of broadcasting and hosting	12.26	*Omited*	*Omited*
D3_Performance	5.73	1.89	*Omited*
D10_Radio and television director	3.95	1.04	*Omited*
Content complexity	3.91	1.83	1.05
D7_Media arts and entertainment studies	3.89	1.27	*Omited*
F/M ratio	3.75	1.5	1.13
D12_Product design	2.03	1.42	*Omited*
Department level	1.44	1.44	1.43
Capital size	1.39	1.37	1.36
D5_Performing arts and film studies	1.27	1.07	*Omited*
Tianjin	1.26	1.26	1.25
Beijing	1.2	1.2	1.15
Business diversity	1.17	1.16	1.16
Chongqing	1.17	1.17	1.16

Shanghai	1.15	1.15	1.14
D8_Fine arts and technology	1.14	1.07	*Omited*
Firm age	1.09	1.09	1.09
D2_Art design	1.08	1.04	*Omited*
Voters	1.07	1.05	1.05
Mean VIF	5.58	1.28	1.17

The dependent variable (DV): Program satisfaction;
N= 1524;

We then re-estimated the regression model using this reduced set of variables (configuration 2). The results are shown in Table S8. Importantly, in this specification, the coefficients for Content complexity, A/V ratio, and their interaction term became more stable and consistent with the study's theoretical expectations. This confirms that removing high-collinearity dummies can yield more reliable coefficient estimates.

Table S8. Regression results after excluding high-VIF discipline dummies

Variable	Model 1	Model 2	Model 3	Model 4	Model 5
_cons	5.172***	5.291***	5.172***	5.292***	5.250***
	(0.128)	(0.145)	(0.128)	(0.145)	(0.146)
Control variables	*Entered*	*Entered*	*Entered*	*Entered*	*Entered*
Dummy variables					
D2_Art Design	-0.012	-0.010	-0.012	-0.010	-0.005
	(0.046)	(0.046)	(0.046)	(0.046)	(0.046)
D3_Performance	0.055†	0.012	0.055†	0.012	0.048
	(0.030)	(0.039)	(0.031)	(0.039)	(0.043)
D5_Performing Arts and Film Studies	0.058	0.051	0.058	0.051	0.058
	(0.038)	(0.039)	(0.039)	(0.039)	(0.039)
D7_Media Arts and Entertainment Studies	0.055**	0.043*	0.055**	0.043*	0.040*
	(0.019)	(0.020)	(0.019)	(0.020)	(0.020)
D8_Fine Arts and Technology	0.011	0.006	0.011	0.007	0.003
	(0.041)	(0.041)	(0.042)	(0.042)	(0.042)
D10_Radio and television director	0.009	0.005	0.009	0.005	0.004
	(0.021)	(0.022)	(0.021)	(0.022)	(0.021)
D12_product design	0.016	0.010	0.016	0.011	0.007
	(0.020)	(0.021)	(0.022)	(0.022)	(0.022)
Independent variables					
Content complexity		-0.034†		-0.034†	-0.021
		(0.020)		(0.020)	(0.021)
A/V ratio			-0.000	0.000	-0.229*
			(0.011)	(0.011)	(0.101)
(Content complexity)×(A/V ratio)					0.084*
					(0.037)
F-Statistics	17.768***	16.967***	16.770***	16.064***	15.562***
Adjusted R-Square	0.158	0.159	0.157	0.158	0.161
∆ Adjusted R-Square	-	0.69%	-1.04%	0.70%	1.47%
Degrees of Freedom	1506	1505	1505	1504	1503

N= 1524;
The dependent variable (DV): Program satisfaction;
Standard errors in parentheses;
*** p<.001; ** p<.01; * p<.05; † p<.1.

Nevertheless, given that the dummy variables are not essential to testing the main hypotheses—and may introduce noise due to redundancy with the A/V ratio—we opted to exclude them from the main analyses. Instead, the main models (Table 4 and Table 5) follow the structure of Model configuration 3 in Table S7, which excludes the entire dummy variable block. This final configuration yields excellent multicollinearity diagnostics, with the maximum VIF at 1.43 and an average VIF of 1.17, providing a stable foundation for regression inference.

4.2. Hierarchical Linear Model

The primary aim of this study is to examine the association between program-level educational characteristics and student satisfaction. Accordingly, the analysis is conducted at the program level (i.e., 1,524 academic programs). However, since many programs are nested within the same university or located in the same region, they may share institutional resources, administrative policies, and cultural environments that influence student satisfaction beyond the program-specific attributes. While the current study does not seek to identify the primary determinants of satisfaction, it is still valuable to explore the multilevel structure of the data. This extension provides a more holistic view of the satisfaction mechanism and offers insights for future research.

To do this, we employed a Hierarchical Linear Model (HLM) incorporating three levels: university, city, and province. Table S9 presents the mixed-effects regression results using Maximum Likelihood (ML) estimation, where all variables from the main model were retained.

Table S9. Mixed-effects ML regression models at the university, city, and province levels

Variable	Model 1	Model 2	Model 3
Constant	5.233***	5.286***	5.242***
	(0.179)	(0.201)	(0.146)
Control variables	*Entered*	*Entered*	*Entered*
Independent variables			
Content complexity	-0.000	-0.039**	-0.041**
	(0.002)	(0.014)	(0.015)
A/V ratio	-0.012	-0.245**	-0.215*
	(0.010)	(0.083)	(0.089)
(Content complexity)×(A/V ratio)	0.005	0.091**	0.080*
	(0.004)	(0.031)	(0.033)
var (Constant)	0.046	0.016	0.002
var (Residual)	0.000	0.031	0.038
ICC	99.4%	33.5%	4.4%
Level	University	City	Province

N= 1524;
The dependent variable (DV): Program satisfaction;
ICC: Intraclass Correlation Coefficient;
Standard errors in parentheses;
*** p<.001; ** p<.01; * p<.05; † p<.1.

The results reveal a striking finding at the university level, where the Intraclass Correlation Coefficient (ICC) reaches 99.4%, indicating that nearly all the variation in student satisfaction is explained by university-level clustering. When university effects are accounted for, the coefficients for Content complexity, A/V ratio, and their interaction become non-significant, although their direction and magnitude remain consistent with those in Table 5.

In contrast, city-level clustering shows a moderate ICC of 33.5%, while province-level effects are relatively small, with an ICC of 4.4%. In both of these models, the coefficients for the two explanatory variables and their interaction remain statistically significant and directionally stable, indicating that the hypothesized effects are robust at broader regional levels.

Interpretation

These findings yield two valuable insights:

First, the overwhelming ICC at the university level (99.4%) suggests that student satisfaction is heavily shaped by university-wide factors. Although CHSI satisfaction data are collected at the program level, our analysis shows that university-level factors—such as shared infrastructure, institutional reputation, and policy frameworks—constitute the dominant source of student evaluations. In contrast, while city- and province-level factors do exert some influence, their impact is significantly weaker. This supports the conclusion that the primary contextual drivers of satisfaction in Chinese higher education are located at the institutional (university) level, not the broader regional or narrow programmatic levels. Although our study does not focus on identifying those contextual drivers, this finding contributes to the broader literature on satisfaction determinants.

Second, the core theoretical framework of this study remains valid. Despite the statistical insignificance of the coefficients under strong university-level clustering, the signs and magnitudes of Content complexity, A/V ratio, and their interaction remain consistent across all three models. This suggests that the hypothesized relationships are robust in structure, even if partially obscured by dominant university-level variance. As our study focuses on program-level educational features rather than institutional or regional differences, the presence of weaker effects in nested models is both expected and reasonable.

4.3. Addressing Potential Endogeneity Concerns

One potential endogeneity concern in this study is whether student satisfaction may be influenced by perceived employment prospects, thereby confounding the observed relationships between content-level features and satisfaction outcomes. Specifically, the worry is that program satisfaction might be driven not only by content characteristics but also by expected employment quality, which could itself be correlated with those characteristics. Although the primary objective of this study is not to identify the dominant drivers

of satisfaction, it is important to assess whether employment-related factors may bias the estimated effects of program content.

To serve as a plausible confounding variable in this context, employment-related perceptions would need to meet the following three criteria:

(1) They must be positively correlated with program satisfaction (the dependent variable);

(2) Negatively correlated with content complexity; and

(3) Negatively correlated with the auditory-visual (A/V) ratio.

Fortunately, we were able to obtain program-level employment satisfaction data from the same source as our primary dataset—the CHSI platform. However, due to differences in data collection procedures and response formats, a direct match was only feasible for 1,000 programs, forming a clean subsample. Because this variable exhibited more than 30% missingness across the full dataset, and given the risk of selection bias, we did not include employment satisfaction as a control variable in the main models. Instead, we conducted a robustness check to evaluate whether employment-related endogeneity poses a serious threat to our conclusions.

Table S10 displays the correlation matrix for the key variables in the 1,000-program subsample, along with their variance inflation factors (VIF). Notably:

(1) Employment satisfaction is weakly but significantly negatively correlated with program satisfaction ($r = -0.096$, $p < 0.001$).

(2) It is positively correlated with content complexity ($r = 0.266$, $p < 0.001$).

(3) It is also negatively correlated with the A/V ratio, although this association is relatively small ($r = -0.093$, $p < 0.001$).

Table S10. Correlation matrix and variance inflation factors (VIFs) among key variables (N = 1,000)

ID	Variables	1	2	3	4	VIF
1	Program satisfaction	1				1.015
2	employment satisfaction	-0.096***	1			1.090
3	Content complexity	-0.062*	0.266***	1		1.083
4	A/V ratio	-0.055*	-0.093***	-0.091***	1	1.018

*Note: ***p < .001, **p < .01, p < .05. All correlations are based on the 1,000-program subsample.*

These findings indicate that employment satisfaction does not meet the criteria for being a valid confounder. Instead of masking or inflating the observed effects, its relationship with the main variables would likely bias the results in the opposite direction, making our observed effects more conservative.

In summary, while employment expectations may intuitively seem like a possible source of bias, the data suggest otherwise. The direction and strength of the correlations indicate that employment satisfaction does not confound the key relationships tested in this study. Thus, we are confident that the main findings

regarding content complexity and auditory-visual modality are not materially distorted by omitted employment-related variables.

Another potential concern is the issue of voter distribution bias. Readers might be worried that certain groups are more proactive in voting while others remain silent. This concern can be alleviated from three aspects: Firstly, the survey has high accessibility. The website is directly operated by the Chinese Ministry of Education, and the questionnaire is comprehensive and mandatory. The website displays over 4.94 million real-name voting records, ensuring impartiality and surpassing most common questionnaire surveys and field research. Secondly, it is highly convenient. After being informed of the mandatory registration, students typically fill out the questionnaire directly on the webpage, which takes only a few seconds, further avoiding feedback bias. Thirdly, based on high accessibility and convenience, the questionnaire results themselves are genuine feedback on real satisfaction. In other words, the impact of satisfaction on reality is based on word-of-mouth effects (Schreiner & Nelson, 2013; Zhen et al., 2019), and the influence of silent groups on reality can be largely ignored.

4.4 Model Effect Analysis

In addition to reporting statistical significance, we offer further interpretation of the magnitude of the effect sizes to enhance the substantive meaning of the findings.

Across both Tables 4 and 5, content complexity consistently shows a statistically significant negative effect on program satisfaction (e.g., $\beta = -0.045$, $p < 0.01$ in Model 2, Table 4). Although the absolute value may appear small, the average satisfaction score in our dataset is 4.69 on a 5-point scale, with a standard deviation of 0.218. Thus, a one-unit increase in content complexity corresponds to roughly 20% of one SD in satisfaction—a substantively meaningful shift in a bounded response scale. This supports the conclusion that more abstract or conceptually demanding content is associated with lower student satisfaction at the program level.

In contrast, the auditory-visual (A/V) ratio has no significant effect when entered alone (Model 3, Table 4), but becomes significant in the presence of an interaction term (Model 3, Table 5). Specifically, in Model 3 of Table 5, the A/V ratio exhibits a negative main effect ($\beta = -0.208$, $p < 0.05$), and the interaction term with content complexity is positive and significant ($\beta = 0.077$, $p < 0.05$). This implies that programs with a higher proportion of auditory content experience lower satisfaction when complexity is high—but that this negative effect is attenuated (i.e., buffered) by the presence of visual modalities. While the interaction coefficient may seem numerically small, it corresponds to a 7.7% shift in satisfaction per unit interaction, which is considerable in educational survey contexts.

Among control variables, Capital size ($\beta \approx 0.005$, $p < 0.05$) and Voters ($\beta \approx -0.088$, $p < 0.001$) show statistically significant effects. However, their magnitudes are relatively small and should be interpreted

cautiously. For instance, the effect of Voters reflects a marginal decrease in satisfaction as program enrollment increases, possibly due to resource dilution or lower instructor-student ratios. Yet the change in satisfaction is less than 0.1 per unit increase in log-transformed voter count, suggesting a statistically significant but modest practical effect.

To summarize, while some coefficients—particularly control variables—are statistically significant with small effect sizes, the main predictors and their interaction demonstrate both statistical significance and educationally meaningful effect magnitudes, especially when interpreted relative to the 5-point satisfaction scale and observed variable distributions. This strengthens the practical relevance of our findings beyond significance thresholds alone.

4.5. On the Explanatory Power of the Model

One potential concern is the relatively modest explanatory power of the models used in this study. Specifically, the adjusted R^2 of the core model—Model 3 in Table 5 of the main text—is 0.161, indicating that approximately 16.1% of the variance in student satisfaction is explained by the model. While this figure may appear low, we offer the following clarifications to contextualize and justify this result:

First, it is important to emphasize that the goal of this study is not to identify the primary determinants of student satisfaction, but rather to explore the overlooked yet theoretically meaningful relationship between educational content characteristics and students' affective evaluations. Existing research consistently shows that student satisfaction is primarily influenced by factors such as teaching quality, career prospects, and interest-discipline fit. High-quality faculty and course design improve learning experiences; strong employment or postgraduate opportunities enhance the perceived value of a program; and alignment with personal interest reinforces identification and satisfaction.

However, our research departs from these well-established determinants and focuses instead on program-level structural and sensory attributes, such as content complexity and auditory-visual modality—variables that are rarely examined in the literature but are critical for long-term discussions on sustainability and inclusiveness in education. Therefore, it is both expected and appropriate that these variables do not account for the majority of variance in satisfaction. Their explanatory power reflects their targeted and theoretically scoped contribution, not their general dominance over all other possible predictors.

Second, the dataset used in this study is exceptionally large and authoritative, covering 73,368 undergraduate students across 1,524 academic programs, from 693 universities located in 198 Chinese cities. The data are collected and verified through CHSI, the official platform administered by China's Ministry of Education, using a real-name registration system, and are fully open to the public. While this lends the dataset unparalleled scale and credibility, it also introduces constraints on variable flexibility. Unlike smaller-scale or self-designed surveys, we were unable to collect detailed individual-level data on known drivers

such as teaching quality, institutional climate, or specific employment outcomes. Many of these variables would likely improve model fit but fall outside the scope or feasibility of our current design.

Looking ahead, future research would benefit from combining large-scale structural data with targeted surveys, thereby bridging institutional features with student-level psychological factors in a more granular way.

Third, despite the overall modest R^2, the contribution of the core independent variables is empirically meaningful. As shown in Table 5:

(1) Adding content complexity and A/V ratio increases the adjusted R^2 by 2.52%;

(2) Adding the interaction term between them contributes an additional 1.51%.

Although these improvements may appear modest, they are nontrivial in the context of real-world, large-scale educational data, especially given that the variables were not specifically tailored to capture satisfaction but rather represent higher-level structural and cognitive features of curricula.

Finally, it is important to note that the entire dataset was preserved in its raw, unfiltered form. No cases were excluded based on distributional characteristics or visual inspection. This choice may have led to a slight underperformance in model fit compared to manually curated datasets, but it also enhances the objectivity and generalizability of the findings. The use of an unmodified, full-sample design constitutes part of the unique value and transparency of this study.

5. Positioning and Limitations of This Study

This study seeks to explore how abstract structural features of academic programs—specifically content complexity and auditory-visual modality—affect student satisfaction in the context of Chinese higher education, particularly in art-related disciplines. Our approach diverges from mainstream satisfaction research, which tends to focus on proximal, individual-level, or institutional service variables (e.g., faculty quality, infrastructure, employment rates, or student-teacher interaction). Instead, this study highlights the value of examining curricular design features and their interaction with cognitive processing mechanisms through the lens of Construal Level Theory (CLT).

5.1 Theoretical positioning: Student satisfaction in the context of art education

This study investigates how program-level structural features—specifically, content complexity and auditory-visual (A/V) modality—affect students' satisfaction within art-related undergraduate programs in China. While it does not directly evaluate outcomes such as creativity or technical skill acquisition, it contributes to the theoretical understanding of how curriculum design influences students' emotional and cognitive responses, which are integral components of sustainable and student-centered education.

In line with Construal Level Theory (CLT), this study frames student satisfaction as a consequence of psychological distance shaped by information abstraction and perceptual modality. More abstract content or predominantly auditory formats may increase psychological distance, thereby weakening affective responses, while more concrete and visually immersive designs may reduce psychological distance and enhance satisfaction. These relationships reflect how students construe and internalize educational experiences at a structural level, offering insight into the cognitive-affective mechanisms that mediate curricular design and evaluative judgments.

Importantly, we do not conceptualize satisfaction as a substitute for learning outcomes such as creativity or competence, but rather as a complementary measure of affective accessibility, engagement, and perceived program fit. In creative education contexts, especially those emphasizing self-expression and aesthetic experience, affective engagement is a critical prerequisite for meaningful learning. Therefore, investigating how program structures influence satisfaction helps extend existing theories of learning by accounting for students' psychological alignment with the curriculum.

Stakeholders who may benefit from these findings include curriculum designers, faculty involved in pedagogical development, and administrators tasked with enhancing student experience and retention. While the study's implications may support institutional strategy and marketing in part, its theoretical contribution lies in illustrating how affective responses to abstract curriculum structures can be systematically modeled and interpreted.

Recent studies have pointed out that increasing knowledge complexity may, under certain conditions, foster higher-order cognition and creative performance. For example, Chae et al. (2015) found that task complexity facilitates knowledge interaction and individual creativity within collaborative teams; Kennet (2024) emphasized the role of layered knowledge structures in stimulating original thinking; and Khalil et al. (2019) highlighted the neural mechanisms through which cognitive complexity enhances creative drives. These insights suggest that educational complexity may serve as a pathway to creativity, particularly when supported by appropriate scaffolding.

While our study focuses on affective responses such as satisfaction, and does not directly assess creativity outcomes, this line of research highlights the importance of contextual and cognitive mediators. In environments lacking sufficient pedagogical or structural support, complexity may result in psychological overload rather than stimulation. Therefore, our findings should not be interpreted as a negation of complexity's creative potential, but rather as a context-bound observation of its affective consequences under current institutional conditions.

5.2 Interpretive boundaries and limitations

This study operates at the structural level of academic programs, relying on publicly available data and expert-coded indicators. Several important limitations should be noted.

First, the A/V ratio is based on aggregated expert evaluations of each program's sensory orientation, rather than on direct observation of teaching practices. It is intended as a proxy for the perceptual emphasis embedded in curricula (e.g., performance- vs. image-based training), but it does not capture the diversity of instructional strategies such as studio-based learning, student-teacher interactions, or project-based assignments that are commonly used in art education. Thus, findings related to A/V ratio should not be interpreted as definitive claims about pedagogy or instructional quality.

Second, the content complexity measure—based on the number and diversity of standardized courses—captures the abstraction level and curricular density of each program. Although this variable was rigorously developed through expert consensus and national syllabi comparison, it does not reflect course-specific variations, teaching styles, or real-time adaptation in the classroom. Therefore, our conclusions pertain to students' affective responses to macro-level program structures, rather than micro-level learning experiences.

Third, the data are aggregated at the program level, and satisfaction scores represent average responses from graduating students across a 16-year period. As such, individual variation, cohort effects, and contemporaneous institutional initiatives are not captured. This limits our ability to make causal inferences or to assess dynamic shifts in teaching quality.

Fourth, the study does not consider a number of well-established drivers of satisfaction—such as teaching quality, career guidance, or institutional culture—not because they are unimportant, but because these factors fall outside the scope of our dataset. This may partially explain the relatively modest R^2 values in our models. Nonetheless, the two core explanatory variables accounted for statistically significant portions of the variance in satisfaction, and their interaction term yielded theoretically meaningful effects consistent with CLT predictions.

Finally, our findings are specific to Chinese higher education in the arts and should be generalized to other national or disciplinary contexts with caution. Future research should explore how content abstraction and modality design function in different educational cultures, and whether similar affective patterns emerge in other creative fields or non-art disciplines.

We suggest using the A/V ratio as a supplementary indicator of the perceived macro-level structure of a curriculum, rather than as a direct proxy for instructional design itself. Its primary function is to reflect the sensory channel preferences of a discipline, thereby aiding in the interpretation of students' psychological response mechanisms.

In summary, this study provides a structural and psychologically grounded account of how students engage with abstract curricula and modality-rich environments in the arts. While it does not evaluate

learning outputs, it offers a novel perspective on affective accessibility and the design of emotionally resonant educational programs.

5.3 Implications for Future Research

This study offers a foundation for future investigations into how the structure and delivery of educational content shape student experiences. Researchers may consider:

(1) Collecting micro-level data through surveys or interviews to triangulate cognitive responses with structural variables;

(2) Incorporating pedagogical design variables, such as project-based learning or assessment diversity;

(3) Testing in other disciplinary contexts, especially STEM or liberal arts, where abstraction and modality function differently;

(4) Examining long-term outcomes, such as persistence, performance, or career satisfaction, to link content design with sustainability in education.

6. Ethics statement

This study utilizes publicly accessible data sources and has been validated and supported by expert interviews in accordance with relevant guidelines and regulations. The following is a detailed explanation of the data sources and their ethical considerations:

Student satisfaction data (dependent variable):

The data on student satisfaction with their programs (program satisfaction) were obtained from the official website of the Chinese Ministry of Education, the "China Higher Education Student Information and Career Center (CHSI)." This platform publishes data in accordance with regulations set by the Ministry of Education, and no ethical approval is required. The data used do not contain any personal or identifiable information.

Course data (independent variable):

The course distribution data for various disciplines were sourced from the "YiFan" website, a platform providing consultation services for the Chinese college entrance examination (Gaokao). Some information on this website requires an account and password to access. This study only uses publicly available or authorized data from this platform, which has been anonymized to ensure that no personal or sensitive information is involved.

Enterprise information data (control variable):

The enterprise information data were sourced from the "TianYanCha" website, a widely used Chinese database of enterprise information. This study only uses aggregated data from this platform, which is publicly available and non-sensitive, and does not involve any ethical concerns.

University official websites data:

The study also consulted publicly available data from the official websites of various universities, including information on course offerings and academic programs. These data are publicly accessible and do not involve any personal or sensitive information, thus no ethical approval is required.

Public information from Baidu search:

Publicly accessible information was also retrieved through Baidu searches, including policy documents, educational reports, and other public data. These are non-sensitive public resources that do not involve any personal or ethically sensitive content.

Expert interviews:

To verify the accuracy and validity of the course distribution data and provide contextual support for the research, expert interviews were conducted with senior professionals in the field of higher education. These experts provided insights based on their professional experience. All interviews were conducted with informed consent from the participants, and no personal or identifiable information was collected or used in the research process.

Since the data used in this study are sourced from publicly accessible resources or involve interactions with professionals who have provided informed consent, no additional ethical approval is required for this research.

References

Alfalah, A. A., & Alganem, S. (2020). The Impact of Construal Level on Happiness, Hope, Optimism, Life Satisfaction, and Love of Life: A Longitudinal and Experimental Study. *Australian Journal of Psychology*, *72*(4), 359-367. https://doi.org/10.1111/ajpy.12297

Brown, R. (2000). Social identity theory: Past achievements, current problems and future challenges. *European journal of social psychology*, *30*(6), 745-778. https://doi.org/10.1002/1099-0992(200011/12)30:6%3C745::AID-EJSP24%3E3.0.CO;2-O

Caballero, A., Fernández, I., Laforêt, B., & Carrera, P. (2024). The Link Between Abstract Thinking Style and Subjective Well-Being: Its Impact When People Are in (Real or Perceived) Financial Scarcity. *The Spanish Journal of Psychology*, *27*. https://doi.org/10.1017/sjp.2024.6

Crouzevialle, M., Schmid, P. C., & Trope, Y. (2023). Beliefs About Abstraction: Low-Level and High-Level Construal Signal Different Lay Theories. *Journal of Experimental Psychology General*, *152*(5), 1351-1367. https://doi.org/10.1037/xge0001332

Hu, J., Qiuyan, Z., Wang, Z., & Tang, S. (2023). Regulatory Focus and Financial Satisfaction: The Sequential Mediating Roles of Construal Level and Opportunity Cost Consideration Among College Students. *Psychology Research and Behavior Management*, *Volume 16*, 2635-2645. https://doi.org/10.2147/prbm.s415053

Park, M., Wu, S., & Funk, R. J. (2024). Regulation and Innovation Revisited: How Restrictive Environments Can Promote Destabilizing New Technologies. *Organization science*. https://doi.org/10.1287/orsc.2022.16770

Petrakis, P. E., & Kanzola, A.-M. (2022). On the Micro-Foundations of Creative Economy: Life Satisfaction and Social Identity. *SUSTAINABILITY*, *14*(9).

Schreiner, L. A., & Nelson, D. D. (2013). The contribution of student satisfaction to persistence. *Journal of College Student Retention: Research, Theory & Practice*, *15*(1), 73-111.

Stets, J. E., & Burke, P. J. (2000). Identity Theory and Social Identity Theory. *Social Psychology Quarterly*, *63*(3), 224-237. https://doi.org/10.2307/2695870

Sun, W., Zheng, Z., Jiang, Y., Tian, L., & Ping, F. (2021). Does Goal Conflict Necessarily Undermine Wellbeing? A Moderated Mediating Effect of Mixed Emotion and Construal Level. *Frontiers in Psychology*, *12*. https://doi.org/10.3389/fpsyg.2021.653512

Wang, Y., & Ieong, S. L. (2019). Will globalized higher education embrace diversity in China? *Frontiers of Education in China*, *14*(3), 339-363.

Zha, Q. (2009a). Diversification or Homogenization in Higher Education: A Global Allomorphism Perspective. *Higher Education in Europe*, *34*(3-4), 459-479. https://doi.org/10.1080/03797720903356628

Zha, Q. (2009b). Diversification or homogenization: how governments and markets have combined to (re)shape Chinese higher education in its recent massification process. *Higher Education*, *58*(1), 41-58. https://doi.org/10.1007/s10734-008-9180-y

Zhen, X., Cai, G. G., Song, R., & Jang, S. (2019). The effects of herding and word of mouth in a two-period advertising signaling model. *European Journal of Operational Research*, *275*(1), 361-373.
